# Exploring non-coding RNA mechanisms in hepatocellular carcinoma: implications for therapy and prognosis

**DOI:** 10.3389/fimmu.2024.1400744

**Published:** 2024-05-10

**Authors:** Yu Tian, Meng Zhang, Li-xia Liu, Zi-chao Wang, Bin Liu, Youcai Huang, Xiaoling Wang, Yun-zhi Ling, Furong Wang, Xiaoqiang Feng, Yanyang Tu

**Affiliations:** ^1^ Research Center, The Huizhou Central People’s Hospital, Guangdong Medical University, Huizhou, Guangdong, China; ^2^ School of Public Health, Benedictine University, Lisle, IL, United States; ^3^ Department of Hepatobiliary Surgery, Affiliated Hospital of Hebei University, Baoding, China; ^4^ Department of Ultrasound, Hebei Key Laboratory of Precise Imaging of Inflammation Related Tumors, Affiliated Hospital of Hebei University, Baoding, Hebei, China; ^5^ Central Laboratory, Hebei Key Laboratory of Cancer Radiotherapy and Chemotherapy, Affiliated Hospital of Hebei University, Baoding, Hebei, China; ^6^ Department of Pathology, The Huizhou Central People’s Hospital, Guangdong Medical University, Huizhou, Guangdong, China; ^7^ Center of Stem Cell and Regenerative Medicine, Gaozhou People’s Hospital, Gaozhou, Guangdong, China

**Keywords:** hepatocellular carcinoma, miRNA sponge, diagnosis and prognosis, therapy resistance, epigenetic factors

## Abstract

Hepatocellular carcinoma (HCC) is a significant contributor to cancer-related deaths in the world. The development and progression of HCC are closely correlated with the abnormal regulation of non-coding RNAs (ncRNAs), such as microRNAs (miRNAs), long non-coding RNAs (lncRNAs), and circular RNAs (circRNAs). Important biological pathways in cancer biology, such as cell proliferation, death, and metastasis, are impacted by these ncRNAs, which modulate gene expression. The abnormal expression of non-coding RNAs in HCC raises the possibility that they could be applied as new biomarkers for diagnosis, prognosis, and treatment targets. Furthermore, by controlling the expression of cancer-related genes, miRNAs can function as either tumor suppressors or oncogenes. On the other hand, lncRNAs play a role in the advancement of cancer by interacting with other molecules within the cell, which, in turn, affects processes such as chromatin remodeling, transcription, and post-transcriptional processes. The importance of ncRNA-driven regulatory systems in HCC is being highlighted by current research, which sheds light on tumor behavior and therapy response. This research highlights the great potential of ncRNAs to improve patient outcomes in this difficult disease landscape by augmenting the present methods of HCC care through the use of precision medicine approaches.

## 1 Introduction

### 1.1 Background of hepatocellular carcinoma

Hepatocellular carcinoma (HCC) is now the second deadliest malignancy worldwide, with an estimated 782,500 new cases and 745,500 deaths in 2012 (1, 2). Patients with early-stage HCC may have a better chance of survival after undergoing procedures such as surgical resection, liver transplantation, local ablation, and other curative treatments (3). Even in patients who have undergone potentially curative treatment for HCC, the 5-year recurrence rate may reach 80%–90%. However, there is a rather high recurrence rate (4). The disease had advanced considerably by the time the majority of patients were identified with HCC (5). The drugs sorafenib and regorafenib, which are small-molecule targeted therapies, have been approved by the United States Food and Drug Administration (FDA) as the standard treatments for the advanced stage of the illness. Although sorafenib is currently the sole conventional first-line systemic therapy option for advanced HCC, the observed median survival duration is only 3 months. If patients with HCC were showing improvement during treatment with sorafenib, regorafenib would be used as a second-line medication. Nevertheless, based on a phase 3 clinical research report, the median survival rate remained at a mere 10.6 months. Although sorafenib and regorafenib have the ability to improve the overall survival of patients with HCC, their length is not excessive. The negative consequences of these drugs and the rise of drug resistance are further sources of increasing anxiety. This highlights the critical importance of seeking out new medicines, with a focus on developing more reliable markers for HCC early diagnosis, treatment, and prognosis. Moreover, hepatitis has been considered as a factor in HCC (6, 7), and therefore, the factors in hepatitis should be understood (8, 9).

### 1.2 Signaling networks in hepatocellular carcinoma

The creation of HCC is caused by the disruption of several signaling pathways, both within and outside the cells (10). Initially, there is a disruption of an intricate network of interconnected pathways that regulate the balance between cell growth and cell death. Cells have the potential to acquire angiogenic, invasive, and metastatic characteristics at the advanced phases of the disease. This is achieved by a process that entails the interactions between neoplastic cells and their surrounding environment. In individuals with HCC, genetic or epigenetic alterations in some elements of the transforming growth factor (TGF)-β pathways, wingless-type (WNT), rat sarcoma virus oncogene (RAS), p53, and retinoblastoma (RB) are frequently observed. These alterations are accountable for the emergence of cancer traits. The activation of the cell membrane receptor tyrosine kinase is caused by several growth factors, the role of which has been demonstrated in chronic liver diseases and the development of HCC (11). Focal growth factor (FGF), hepatocyte growth factor (HGF), platelet-derived growth factor (PDGF), transforming growth factor-beta (TGF-α), epidermal growth factor (EGF), and vascular endothelial growth factor (VEGF) are the molecules that activate these pathways. In the end, these substances boost cell survival and proliferation by attaching to specific receptors on cell surfaces. Angiogenesis, invasion, and metastasis are all processes that they initiate. The met proto-onco-gene (MET), also known as the HGF receptor, was overexpressed in 40%–70% of HCCs (12–15). Moreover, it has been discovered that there is an excessive amount of VEGF present, as evidenced by studies (16, 17), and this has been linked to the development of an advanced cancer phenotype (18–22). There was a correlation between angiogenic and invasive phenotypes and FGF overexpression (23–25). It was discovered that this was true when combined with VEFG. According to some research, overexpression of PDGF and its receptor may contribute to the development of HCC (26, 27). Regarding downstream effectors, it has been established that HCC is marked by RAS overexpression (28), while activating point mutations have only been found extremely infrequently (29, 30).

The Wnt/β-catenin pathway is one of the additional signaling pathways usually involved in hepatocarcinogenesis (31). The stability of β-catenin, which allows its translocation to the nucleus, is the outcome of its activation. It binds to TCF/LEF transcription factors, which are particular to T cells, at that spot. Metalloproteases, cyclin D1, VEFG, c-myc, and c-met are only a few of the numerous target genes that these transcription factors activate. Several mechanisms have been identified as responsible for the aberrant activation of the Wnt-β-catenin pathway in HCC. Approximately 12%–26% of HCC exhibit mutations at the N-terminus of β-catenin that result in increased functionality. Additionally, HCCs may also experience deletions, mutations, or epigenetic changes in the E-cadherin gene. Furthermore, approximately 8%–13% of HCCs are characterized by mutations that lead to a loss of function in the AXIN1 or AXIN2 genes (32, 33). This stability of β-catenin could be explained by the fact that growth hormones phosphorylate and then deactivate GSK-3β, in conjunction with these alterations. Some examples of growth factors are insulin, insulin-like growth factor (IGF)-1, FGF-2, EGF, PDGF, HGF, TGF-β, and tumor necrosis factor (TNF)-α. Another possible reason for the stability of β-catenin could be the use of the Erk-priming mechanism or the activity of the HBV-X protein to promote GSK-3Ω inhibition (34). Recent investigations have revealed that hepatic adenomas are characterized by mutations in the β-catenin gene. These modifications are associated with an increased likelihood of developing cancerous changes (35).

There are a number of downstream effectors that are abnormal in cancer, in addition to the primary consequences of signal transduction pathways, which include cell proliferation and survival. For instance, there has been a lot of study on Rb1, cyclins, CDK inhibitors, cyclin-dependent kinases (CDKs), and their coordination in regulating the cell cycle in HCC. A loss of chromosome 13, found in approximately 30% of HCCs, is one possible mechanism by which the RB1 gene is rendered inactive in these tumors (36, 37), or epigenetic processes (38). Point mutations are very rare. The RB1 protein may be rendered inactive due to an aberrant synthesis of Gankirin, a protein that can bind to RB1, enhance RB1 phosphorylation, and ultimately lead to its proteosome complex fragmentation. It was found that Gankirin was upregulated in all HCC examples (39). In addition, compared to the parenchyma around the tumor, HCC tissue often had reduced levels of cell cycle negative regulators, whereas cyclins were upregulated among the components that control the cell cycle (31). Approximately 60% of HCC exhibit an elevated level of cyclin D1/CDK4 (40). Among the CDK inhibitors, p16/lNIK4A, which targets CDK4 and CDK6 specifically and inactivates CDK4/cyclin D1 complexes (41), is functionally inactivated in a significant portion of HCCs. This is because deletions at the short arm of chromosome 9 occur in approximately 20% of HCCs (32, 36, 37) and methylation of the p16/INK4A promoter occurs in 30% to 70% of cases (42, 43). The CDK inhibitors of the CDK interacting protein (KIP) family, more especially CDKN1B/p27 and CDKN1C/p57, are two more proteins that play a role in HCC tumor suppression and direct cell cycle regulation. Research has demonstrated that compared to the surrounding cirrhosis, HCC tissue exhibits reduced expression levels of CDKN1B/p27 and CDKN1C/p57 (44). Twenty to fifty percent of HCCs show a lack of maternal allele methylation at the KvDMRI imprinted locus at eleven p15.5, where the CDKN1C/p57 gene is located. This has been previously confirmed by us and other researchers. Reductions in CDKN1C/p57 expression have been associated with this mechanism (45–48). The overexpression of miR-221/222, which is found in approximately 70% of HCCs, is one of the main processes that lead to the downregulation of p27 and p57 molecules, as will be shown in the next paragraphs (49–51). Conversely, a high p27 expression level is not always associated with a low cell proliferation rate in head and neck malignancies. Evidence suggests that sequestration into complexes containing cyclinD1 and CDK4 renders p27 inactive in such contexts (52). The current review focuses on understanding the epigenetic modifications in HCC, specifically in relation to non-coding RNAs (ncRNAs), in order to gain insight into the dysregulation of molecular pathways and signaling networks.

## 2 miRNAs in hepatocellular carcinoma

### 2.1 miRNA biogenesis and interaction with molecular pathways

**Figure 1:**
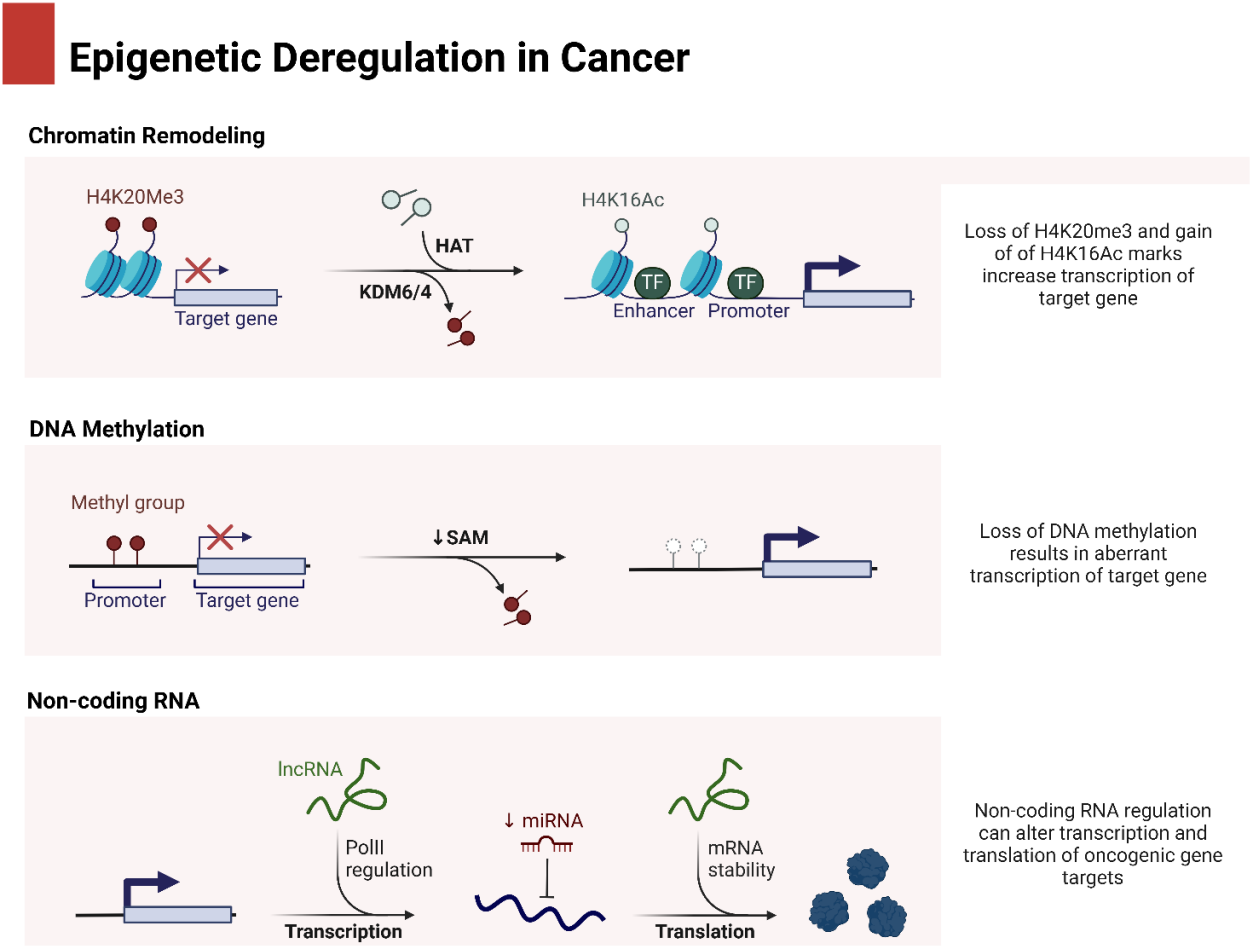
An overview of epigenetic dysregulation in cancer.

In the process of maturation and decay of RNA molecules, the enzymatic degradation of double-stranded RNA is critical (53). MicroRNAs (miRNAs) are small RNA molecules with one strand that are synthesized naturally by living things. They include approximately 22 nucleotides (54). The miRBase database, accessible at http://www.mirbase.org, now contains 1,492 registered human miRNA sequences. miRNAs, despite lacking coding sequences, have been demonstrated to have a role in the post-transcriptional control of genes that are crucial for fundamental cellular processes and diseases (55). The miRNA gene is typically translated by RNA polymerase II in the nucleus, leading to the formation of a primary transcript called pri-miRNA. This transcript is often between one and four kilobases long (56). Monocistronic transcripts refer to transcripts that contain only a single miRNA gene located downstream of a promoter. On the other hand, the miR-17-92 miRNA polycistron serves as an illustration of a polycistronic transcript, which originates from a single transcript that groups together many miRNA gene products (57). Approximately 50% of miRNA genes, specifically intragenic or mirtrons, are believed to be regulated by the host gene. Intergenic areas are potential sites for them, indicating that they are probably distinct transcriptional units (58). The percentage that changes depends noticeably on where the miRNA genes are located in the genome. The protein DiGeorge syndrome critical region gene 8 (DGCR8) and enzyme nuclease Drosha then come together to form the microprocessor complex. This complex is responsible for converting pri-miRNA, or primary miRNA, into pre-miRNA, or precursor miRNA. It is Exportin-5’s job to deliver the approximately 70-nucleotide pre-miRNA to the cytoplasm. When the miRNA–miRNA duplex reaches its target, another nuclease called Dicer breaks it down into smaller pieces of approximately 18–25 nucleotides (59). **Figure 1** is an overview of epigenetic dysregulation in human cancer.

miRNAs demonstrate interaction with various molecular factors in cancer. The miRNAs can dually increase or decrease tumorigenesis, highlighting their function as a double-edged sword. miR-429 impairs the progression of endometrial tumor, and through regulation of DDX53, it enhances drug sensitivity (60). miR-21 and miR-125b are considered as biomarkers in ovarian cancer with overexpression. Moreover, downregulation of miR-125b causes platinum resistance in ovarian cancer (61). miR-1299 is another factor inhibiting cervical tumor. miR-1299 has low expression in cervical cancer, and it is suppressed by KCNQ1OT1 (62). In thyroid cancer, miR-1284 stimulates apoptosis, while it reduces growth and metastasis of tumor cells through E-cadherin upregulation and downregulation of N-cadherin (63). More importantly, miRNAs can develop feedback loop with their targets such as the loop between PAX5 and miR-142 in breast tumor to modulate expression levels of DNMT1 and ZEB1 (64). The plasma levels of miR-1290 and miR-29c-3p in lung cancer are biomarkers (65). Moreover, miR-629 downregulates LATS2 to enhance the growth of prostate tumor (66). The expression of miRNAs can also be regulated by exosomes in human cancers, since the exosomes are able to transfer miRNAs (67). The downregulation of FXYD5 by miR-1180 can suppress the migration and metastasis of pancreatic tumor (68).

**Figure 2:**
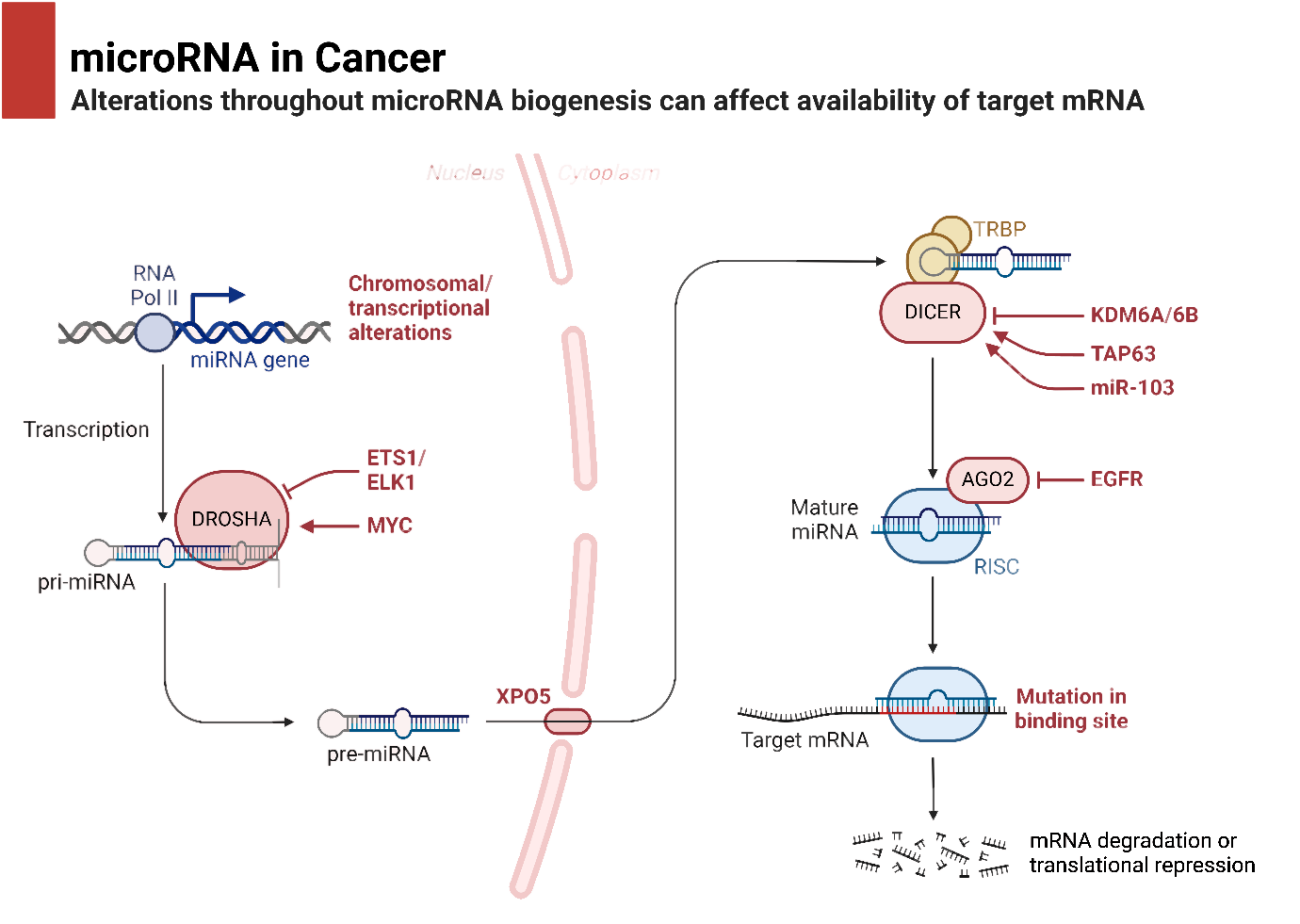
miRNA biogenesis and its function.

It is common practice to remove the miRNA passenger strand before loading a mature miRNA guide strand into the RNA-induced silencing complex (RISC). The extent to which this complex regulates a gene depends on how well the miRNA and its target mRNA sequence complement one another in the 3′-untranslated region (3′-UTR). For RISC to cleave and remove mRNA, the mRNA and RISC must be highly complementary. Conversely, if the complementarity is not ideal, it will hinder the translation process (69). Lower levels of messenger RNA (mRNA) were demonstrated to precede a decrease in protein levels in 84% of the cases (70). Functional mRNA target sites typically consist of six or seven nucleotides and exhibit complementarity to miRNA sequences. The sequence is succeeded by an adenosine, which is commonly known as the “seed” sequence of the miRNA. miRNAs that are targeted for destruction are eliminated in cytoplasmic processing bodies, sometimes referred to as P-bodies, which are situated in the cytoplasm (71). The discovery of additional ncRNAs in the last several years deserves note. Possible functions for these RNAs include regulation of gene expression or association with hepatocellular cancer. The conversion of the short nucleolar RNA (snoRNA) ACA45 into RNAs that bind to Argonaute proteins (Ago) and are 20 to 25 nucleotides long by Dicer provides support for the research conducted by Ender et al. Furthermore, luciferase reporter tests provided evidence that ACA45 displayed miRNA-like functionality (72). Yang and colleagues uncovered a long non-coding RNA (lncRNA) known as High Expression in Hepatocellular Carcinoma (lncRNA-HEIH). The expression of this lncRNA is favorably linked to the recurrence of tumors and negatively correlated with survival. Additionally, its expression is changed in HCC. In addition, they demonstrated that the use of shRNA to downregulate lncRNA-HEIH can greatly reduce tumor growth in a mouse model obtained from xenografts (73). **Figure 2** is an overview of miRNA biogenesis along with its function in cancer.

The low 5-year survival rate can be linked to two primary factors: a high rate of recurrence and resistance to treatment. The mortality rate is below 10%. Surgical resection is the most effective treatment for HCC; nevertheless, only a small proportion of patients, namely, 10%, are suitable candidates for this therapy at the time of their initial diagnosis. Conversely, the likelihood of survival after removal is approximately 70% if the tumor is solitary and smaller than 2 cm in size. Therefore, obtaining an early diagnosis is imperative in order to improve the prognosis. While AFP (>400 ng/mL) is commonly utilized as a biomarker for breast cancer, its sensitivity and accuracy are very moderate, and it fails to detect HCC in 50% of patients. Also known as prothrombin induced by vitamin K absence-II (PIVKA-II), another marker is referred to as des-γ-carboxy prothrombin (DCP). The aberrant and functionally inactive form of prothrombin is clearly recognizable due to its lack of N-terminal carboxylation prior to release (74, 75). The carboxylase, which typically performs this function, is frequently missing in HCC cells. By combining these biomarkers with AFP and AFP-L3, it is possible to provide a more accurate prediction of the course of HCC in individuals with chronic HBV or HCV. This is particularly useful for assessing portal vein invasion and intrahepatic metastases (75, 76). However, it is important to note that DCP elevation might be caused by other reasons, and a normal DCP level does not always exclude the presence of HCC. Therefore, it is imperative to possess supplementary biomarkers, namely, those associated with first changes, in order to improve the prognosis.

Dysregulation of miRNAs plays a prominent role during hepatocarcinogenesis. miRNA profiles can differentiate between the general population and patients with HCC, as well as other liver illnesses (54, 77). There is a clear difference in miRNA profiles between normal and cancerous tissues, and these differences may even vary by subtype of cancer (78). The presence of miRNAs in liver tumor tissue, serum, plasma, and urine raises the prospect of a less invasive way to track the treatment’s efficacy and predict the prognosis. Several studies have found a connection between miRNAs and HNS cancer. Higher levels of miR-17-92, miR-21, miR-221, miR-222, and miR-224 are frequently observed in HCC tumors (54, 79). In contrast, miR-199a and miR-199b are often downregulated together with let-7, miR-200, miR-29, miR-122, miR-123, and miR-199b (54, 78, 80). Despite miR-122 being downregulated in initial HCC tumors, it is upregulated in the serum of patients with HCC (81). The secretion of miR-122 into the bloodstream from tumors could be the reason behind this. However, miR-199 is highly expressed in healthy liver tissue, despite its downregulation in HCC (82). The downregulation of miR-199a/b is linked to poor survival rates. This is due to the fact that miR-199a/b-3p reduces HCC by blocking the pathways that involve p21-activated kinase 4 (PAK4), Raf, MEK, and ERL. As in the previous instance, miR-99a downregulation is linked to a bad prognosis (83). In contrast, miR-224 is upregulated in HCC (84), and newer research shows that it reflects tumor stage and liver function; greater levels are associated with a worse prognosis (85). Regional variations in the etiology of HCC may impede the creation of effective biomarker panels. Changes in miRNA expression that are linked to the cause can further hinder these efforts. A recent study utilized miRNA expression profiling of liver tissue to identify dysregulated miRNAs that are linked to HBV or HCV-HCC (86). Out of 40 miRNAs, 12 showed dramatically dysregulated expression. All six of these were confirmed in tissue samples; however, plasma levels of miR-126 and miR-142-3p were shown to be elevated in patients with HBV with HCC compared to those without HCC. Both miRNAs improved the area under the curve (AUC) to 0.92 when added to AFP, but neither of them was more effective than AFP alone. Patients with HCV-related HCC and non-viral HCC did not differ in miR-126 levels. This discovery raises the possibility that miRNA biomarkers’ predictive effectiveness can be affected by changes in the underlying causes of HCC. The prevalence of HBV-related HCC is higher in Asia and other regions with a large HBV population, but the reverse is true in Japan. Despite the fact that vaccines are decreasing the frequency of HBV, non-alcoholic steatohepatitis and other non-viral causes of HCC are increasing (87). Thus, it is necessary to assess biomarkers that have been developed and confirmed in one area in other places that have different underlying causes.

### 2.2 Expression of miRNAs and function in HCC development

A considerable number of investigations have revealed that miRNAs can affect basic cellular functions including differentiation, proliferation, death, invasion, and metastasis (88, 89). Tumors may exhibit distinct miRNA expression patterns compared to normal tissues. These profiles also vary based on the type of tumor that is present. It is noteworthy because miRNA directly targets protein-coding genes related to the cell cycle, death, and metastasis in HCC (90). A recent microarray study has demonstrated that a specific subset of miRNAs undergoes both upregulation and downregulation throughout the progression of HCC. miRNAs are frequently shown to be reduced in their expression in HCC. The downregulated miRNAs may target oncogenes, indicating their possible role in promoting cancer. In contrast, certain miRNAs that are elevated play a role in the progression of cancer in HCC and may be targeted by genes that inhibit tumor growth.

A variety of chronic liver illnesses have been associated with miRNAs, such as alcoholic liver disease, viral hepatitis, non-alcoholic steatohepatitis (NASH) (91–93), and alcoholic liver disease (94–96). On top of that, miRNAs are involved in the onset of HCC and these other chronic diseases. The accumulation of ethanol-induced hepatocyte fat occurs during alcoholic liver disease because miR-217 suppresses the expression of SIRT1 (97). In alcohol-induced HCC, the expression of the miRNAs miR-126, miR-27b, miR-182, miR-183, miR-199, miR-200a, miR-214, and miR-322 was reduced (79, 96). Patients with alcoholic steatohepatitis had a decrease in miR-27 due to epigenetic changes brought about by alcohol usage (98). The risk of HCC is increased in both the onset and progression of non-alcoholic fatty liver disease (NASH), and miRNA is involved in both stages. Recent research has linked miRNAs to the activation of hepatic stellate cells (HSCs), which, in turn, contribute to the advancement of non-alcoholic fatty liver disease (NASH) (99). An increase in the expression of miRNA-33a and sterol regulatory element-binding protein-2 (SREBP2) resulted in the accumulation of unbound cholesterol. This was achieved by activating HSC and interfering with the SREBP2-mediated cholesterol-feedback pathway in HSC, while also inhibiting the signaling of peroxisome proliferator-activated receptor-γ (99). Overexpression of miRNA-21 causes a decrease in expression of tumor suppressor phosphatase and tensin homolog (PTEN) in hepatocytes when unsaturated fatty acids are present (91). The miRNA miR-155 has the ability to decrease the expression of another tumor suppressor gene known as CCAAT-enhancer-binding protein-β. In addition, mice fed a diet low in amino acids including choline had elevated levels (92, 93).

Hepatitis B virus (HBV) and hepatitis C virus (HCV) infections are the leading causes of cirrhosis and HCC (100). Cirrhosis of the liver is linked to a gradual risk of developing liver cancer, known as hepatocarcinogenesis, which ranges from 5% to 30% within a 5-year time frame (100). The experimental model revealed that just two specific miRNAs, miR-210 and miR-199-3p, had a discernible impact on the expression and replication of the HBV gene in individuals with HBV infection. The life cycle of HBV is indirectly controlled by several cellular miRNAs, which impact cellular proteins related to the virus (101). Ura and collaborators studied the expression of 188 miRNA in HCC and the adjacent normal tissues obtained from 12 patients with HBV infection and 14 patients with HCV infection. The expression of six miRNAs was shown to be downregulated in patients with HBV, while the expression of 13 miRNAs was observed to be downregulated in patients with HCV (102). These findings suggest that the miRNA profiles of HBV and HCV infections differ significantly in terms of pattern. The presence of miR-96 or miR-26 is dramatically increased in tissues linked to HBV-related colon cancer, according to multiple studies. Using sequencing techniques, Takizawa and colleagues were able to uncover whole miRNA profiles from 314,000 trustworthy reads collected from HCC tissue and over 268,000 credible reads obtained from the nearby normal liver. These miRNA profiles were constructed using tissues from both HCCs and adjacent normal livers. A study conducted using bioinformatics discovered that HCC was associated with changes in miRNAs such miR-122, miR-21, and miR-34a (103). Given this information, further investigation into the specific miRNA associated with HBV-related HCC could lead to the creation of a viable therapeutic tool for patients with HCC who have HBV infection. Furthermore, miR-196 has a crucial function in HCV-related non-small cell lung cancer by suppressing Bach1, a mammalian transcriptional repressor with a basic leucine zipper structure, and elevating the expression of hemeoxygenase 1, a transcriptional regulator. Furthermore, Diaz and co-workers found that among a total of 2,226 human miRNA, a specific subset of 18 miRNA exhibited expression only in HCV-related HCC (104). One of these 18 miRNAs has been recognized as being the cause of HCC and is correlated with networks that involve retinoic acid, p53, and PTEN (101, 105). The provided findings declare that miRNA pathways have a noticeable influence on the development of HCC during HCV infection.

The miRNAs have shown significant interaction with other molecular pathways in HCC. miR-21-5p has been shown to induce sorafenib resistance in HCC, and through SIRT7 inhibition, it increases USP24 expression to accelerate tumorigenesis (106). The regulation of lipid synthesis and uptake by the miR-3180 can suppress the progression of HCC (107). The enrichment of miRNAs in the exosomes can affect HCC. Exosomal miR-200b-3p has been shown to suppress ZEB1 in increasing M2 polarization of macrophages and stimulation of the JAK/STAT axis (108). On the other hand, miR-424-3p has been shown to enhance the invasion and migration of HCC through reducing the activation of SRF on STAT1/2 (109). miR-17-5p downregulates TGFβR2 to impair the progression of HCC (110). miR-4270 suppresses DNMT3A-induced methylation of the HGFAC promoter to disrupt the progression of HCC (111). The increase in the levels of caspase-3 and caspase-9 is vital for reduction in the viability of tumor cells. miR-767-3p can downregulate caspase-3/-9 to enhance tumorigenesis in HCC (112).

### 2.3 Circulating miRNAs in HCC development and diagnosis

miRNAs can enter the bloodstream through two distinct mechanisms: passive diffusion, which does not require energy, or active transport, which is selective and occurs in response to certain stimulants (113). The initial stage occurs during cellular disruption in pathological conditions, such as tissue damage, and does not necessitate the presence of adenosine triphosphate (ATP). The active secretion of miRNA is contingent upon the presence of ATP and the temperature, regardless of cellular stimulation. This is the main mechanism by which circulating miRNA (cmiR) is generated for circulation. Microvesicles (MVs) and apoptotic bodies are two potential methods of encapsulating cmiR. In addition, cmiR can be identified using multiproteins or lipoprotein-binding miRNA complexes (114, 115). The conveyance of cmiR is aided by both the aforementioned structures. Based on the findings, Ago 2 is responsible for overseeing the process by which miRNA is loaded into exosomes. Most cell types have the ability to release exosomes, which can be obtained from various physiological fluids such as serum and urine. Given the large quantity of miRNA found in exosomes, it is extremely probable that exosomal miRNA might be used as significant markers for the detection of diseases (116). A certain group of plasma miRNAs does not contain exosomes or MVs. These miRNAs are protected and made stable by binding to Ago 2, Nucleophosmin 1, or high-density lipoprotein (117). The aforementioned research demonstrates that cmiR is both stable and readily obtainable, making it a viable biomarker for the identification of illnesses. Moreover, the expression of miRNA in tumor tissues may not necessarily correspond to its expression in the blood. As a result, tumor-derived miRNA was conceived. The expression of these miRNAs was found to be elevated in tumor tissues and the bloodstream, but decreased dramatically in the bloodstream after tumor excision. Thus, cmiR plays a pivotal role in monitoring the onset and recurrence of tumors. Budhu et al. propose that identifying a specific miRNA signature linked to metastasis could aid in the early identification of HCC (118). This indicates that the dysregulation of miRNA may have diagnostic value. Furthermore, several studies have shown that miRNA signatures may have additional clinical significance in relation to HCC (119, 120). A number of clinical studies have shown that miRNAs are biomarkers for patients during chemotherapy (NCT03779022) and circulating miRNAs can be utilized for the development of diagnostic factors in brain cancer (NCT03630861). The miRNAs are enriched in serum and plasma of ovarian cancer (NCT06329323). For HCC, miRNAs are also potential diagnostic factors based on clinical studies. The hepatic and circulating miR-221 and miR-222 in HCC have clinical significance (NCT02928627). Moreover, in Somali patients, miRNAs are diagnostic factors in HCC (NCT03227510).

### 2.4 miRNAs and drug resistance in HCC

At present, the pharmacological therapy for HCC includes targeted treatment, chemotherapy, and immunotherapy. Sorafenib, regorafenib, lenvatinib, and tivantinib are drugs that specifically target molecules at the molecular level. Adriamycin, 5-FU, cisplatin, and oxaliplatin are often prescribed chemotherapy drugs in clinical settings. Immunotherapy is a novel approach for the treatment of HCC. The treatment mostly consists of monoclonal antibodies and immune checkpoint inhibitors that target the cytotoxic T lymphocyte antigen-4 (CTLA-4) receptor, programmed cell death protein 1 (PD-1), and PD-1 ligand (PD-L1). This treatment utilizes immunotherapies such as nivolumab, pembrolizumab, MED14736, ipilimumab, and tremelimumab, among other options. miRNAs are short, single-stranded ncRNAs that consist of 19–24 nucleotides. miRNAs have the ability to attach themselves to the 3′-UTRs of specific mRNAs and inhibit their translation or synthesis. miRNAs are involved in various cellular processes such as differentiation, proliferation, death, angiogenesis, and metabolic stress responses (121–123). In the development and advancement of numerous cancers, including HCC, studies have revealed that dysregulated miRNAs can function as tumor suppressors or oncogenes (124–126). It should be noted that compared to drug-sensitive cells, many drug-resistant HCC cells exhibit drastically different miRNA expression. More tailored HCC treatments may be possible as a result of this finding, which opens the door to the potential use of several miRNAs for medication efficacy prediction (127–130). The expression of immune checkpoint molecules in tumor microenvironments can be controlled by miRNAs (131). However, the precise mechanism by which miRNAs contribute to the development of resistance to immune checkpoint blockers remains incompletely known. In addition, there is emerging research suggesting that other miRNAs may play a role in sorafenib resistance. However, only a limited number of miRNAs have been identified to have a role in developing resistance to other advanced targeted treatments.

Sorafenib, an orally administered multikinase inhibitor, was initially demonstrated to decrease cell growth and blood vessel formation by specifically targeting BRAF, Raf-1, Flt3, VEGFR-2/3, and PDGFR-β. Moreover, it was found that sorafenib possesses the capability to selectively affect signaling pathways that are not reliant on Raf, specifically those involved in regulating apoptosis and cell cycle progression (132–134). Sorafenib, an FDA-approved conventional targeted therapy medication for lung cancer, has demonstrated survival benefits in patients with advanced HCC globally. However, the majority of individuals later developed problems that require treatment resistance. Sorafenib resistance is currently caused by various pathways, such as microenvironmental hypoxia, epithelial–mesenchymal transition (EMT), cellular stem cell activation, resistance to apoptosis, autophagy, cell cycle dysregulation, c-jun and/or AKT activation, and abnormal expression of miRNAs and lncRNAs (130, 135–141). Several cancer-causing miRNAs have the ability to induce resistance to sorafenib (139, 142–145). Sorafenib resistance could be caused by miRNAs such miR-93, miR-216a, and miR-217, which target cell cycle protein-dependent kinase 1A (CDKN1A) and impact apoptosis and TGF-β signaling (143, 144). In addition, miR-181 may target and decrease Ras association domain family member 1 (RASSF1), which could lead to sorafenib resistance (145).

Adriamycin, an antibiotic of the anthracycline class, is a powerful inhibitor of DNA and RNA synthesis in rapidly dividing tumor cells. It is a non-specific periodic medicine. Adriamycin has the capability to permeate into the nucleus of HCC cells, where it can potentially engage with DNA and ultimately induce apoptosis. Multiple mechanisms implicated in the development of adriamycin resistance in HCC have been discovered (146). Oncogenic miRNAs can enhance resistance to adriamycin. There are scientific data indicating that Let-7a has the ability to enhance the resistance of HepG2 cells to the antibiotic adriamycin (147). Furthermore, it has been found that the external production of miR-519d specifically affects numerous tumor suppressor genes, including p21 and PTEN. This ultimately leads to an enhanced resistance of HCC cells to adriamycin (148). In contrast, research has shown that numerous tumor suppressor miRNAs can effectively overcome adriamycin resistance in HCC. For instance, a total of 30 separate HCC tissues exhibited a substantial decrease in the expression of MiR-26a/b compared to normal tissues. Moreover, research has discovered that elevated levels of externally introduced miR-26a/b can augment the responsiveness of HCC cells to adriamycin. This is achieved through the specific targeting of ULK1 expression and the autophagy signaling pathway, both in laboratory settings (*in vitro*) and in living organisms (*in vivo*) (149).

### 2.5 miRNA delivery in HCC therapy

**Figure 3:**
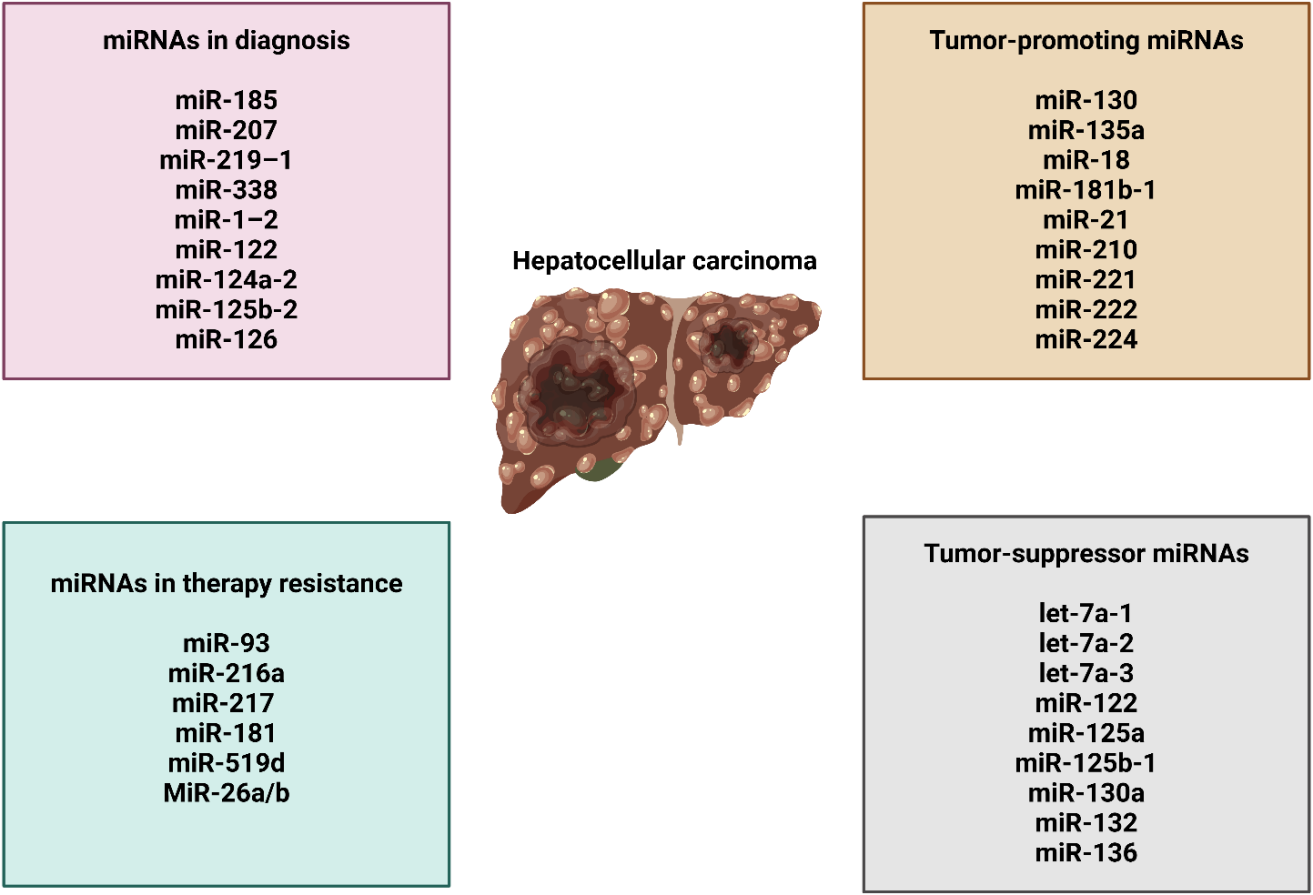
The function of miRNAs in HCC, their association with therapy resistance, and their role in cancer diagnosis.

Several miRNAs possess the capacity to be utilized in clinical contexts for the therapeutic management of HCC. However, there is still a dilemma about the method of delivering medicinal chemicals in a more concentrated form to certain tissues (150). The intricate network of hepatocytes and non-parenchymal cells, along with the non-specific uptake of therapeutic vesicles, is the primary challenge that needs to be addressed in order to effectively administer miRNA for HCC treatment. Research has been conducted on non-viral distribution mechanisms. An important advantage of a non-viral strategy is the ability to exert precise control over the functionality of therapeutic medications. Conversely, the drawback of non-viral delivery is that its effects are transient and limited in duration after a certain number of administrations. This limitation arises from the fact that therapeutic miRNAs are susceptible to systemic excretion or degradation (sometimes referred to as degradation). miRNAs can be incorporated into stable nucleic acid lipid particles, or SNALPs, due to their capacity to inhibit rapid disintegration. Coating these SNALPs with polyethylene glycol (PEG) increases their circulation period (151, 152). Increased miRNA stability and off-targeting effects are both brought about by 2′-O-methyl modifications (153). As a result of the inherent viral tropism, virus-like particles (VLPs) can also be utilized for the purpose of distribution. Nevertheless, due to the fact that VLP is closely related to a viral external protein, it has the potential to trigger the immune response (154). Moreover, investigations on miR-375 have illustrated that this miRNA is more effectively delivered to the liver when it is conjugated to cholesterol (153). Nevertheless, as previously stated, the therapeutic effect of miRNAs transferred through non-viral means is only transient. Therefore, it is preferable for miRNAs to exhibit consistent and enduring efficacy in the treatment of chronic and inherited illnesses (155). RNAi sponges are responsible for delivering RNA strands that can be cleaved, hence preventing the degradation of siRNA during transportation. Moreover, the application of RNAi sponges presents a distinctive therapeutic approach due to their capacity to effectively inhibit the target miRNAs in a dominant fashion (156). HepG2 cells that were genetically modified with an adenoviral vector containing a miR-21 sponge experienced a significant decrease in the synthesis of miR-21, resulting in a reduction in the expression of MAP2K (157). Preclinical experiments have successfully utilized viruses to deliver miRNAs for the treatment of HCC. For example, when miR-26a was injected into HCC mice via AAV through the bloodstream, it showed a notable ability to inhibit tumor growth (158). It is not surprising that viral vectors have the ability to provide therapeutic effects that continue for a long time. The clinical safety of these vectors affects their use in clinical settings. The liposome technology known as Smarticles^®^, licensed from Marina Biotech, Inc (159), is utilized for the delivery of MRX34, the initial miRNA mimic currently undergoing clinical trials. At a neutral pH, Smarticles^®^ possess an anionic charge, whereas in an acidic environment, they acquire a cationic charge. Because tumors have a lower pH, their absorption is enhanced while the risk of adverse interactions with normal tissue is reduced. The liposome component of MRX34 consists of different combinations of palmitoyl oleoyl phosphatidyl choline, dioleoyloxytrimethylammonium propane, 1,2-dimyristoylglycerol-3-hemisuccinate, and cholesterol. These mixes are used to inhibit the degradation of miR-34 and improve its distribution efficiency. **Figure 3** provides a comprehensive summary of miRNAs in HCC.

## 3 Long non-coding RNAs in hepatocellular carcinoma

### 3.1 Function of lncRNAs and their interaction with molecular pathways

**Figure 4:**
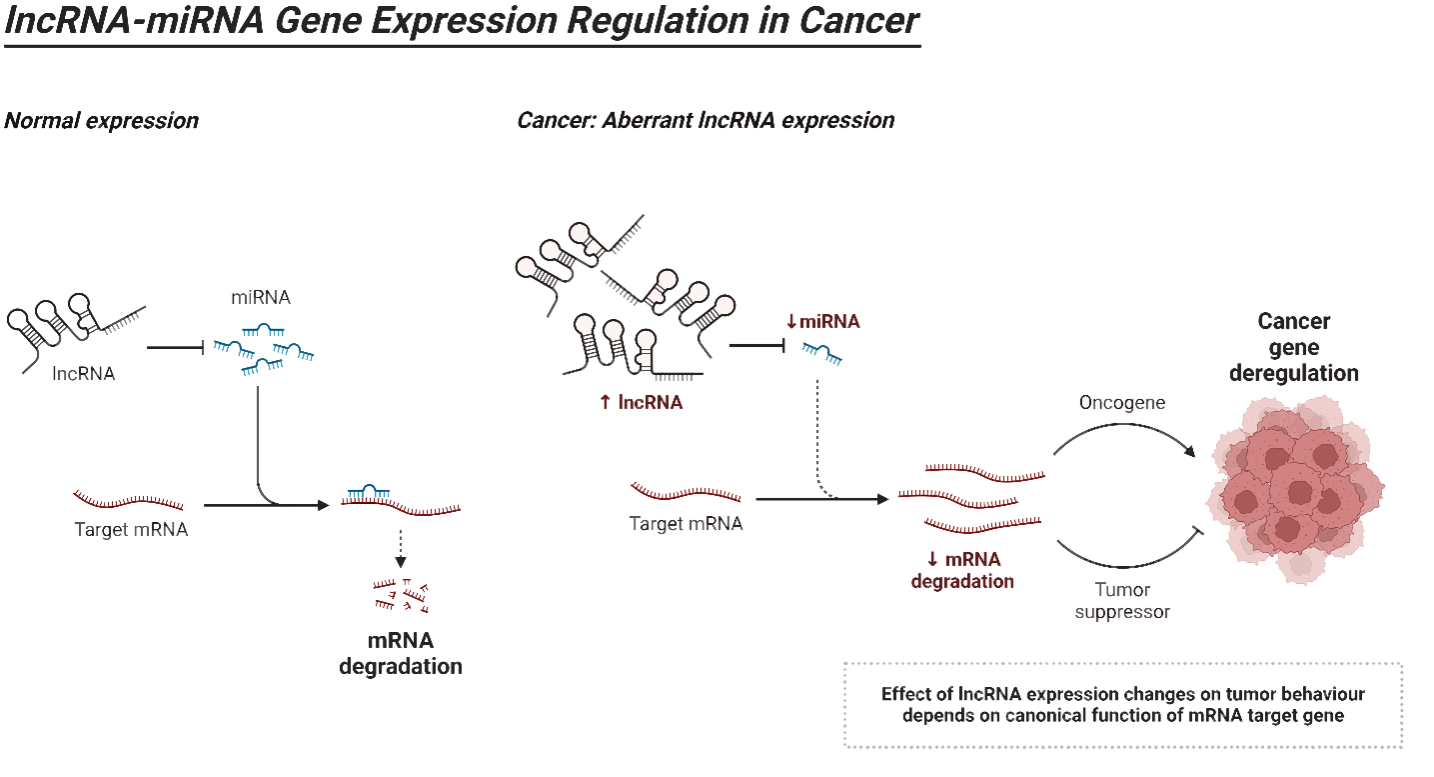
An overview of lncRNAs.

LncRNAs can be classified into five distinct categories according to their proximity to neighboring protein-coding genes: (i) In the sense form, lncRNAs are located in the same region as the sense strand of a protein-coding gene. (ii) In the antisense form, lncRNAs are located in the same region as one or more exons of a protein-coding gene on the opposite strand, and they start at the 3′ end of a protein-coding gene. (iii) In the bidirectional form, both the lncRNA and the protein-coding gene on the opposite strand are expressed within a close distance of less than 1,000 base pairs (bp) from each other in the genome. The intron form of lncRNAs refers to cases when lncRNAs start within an intron of a protein-coding gene, without overlapping exons. On the other hand, the intergenic form, also known as large intervening non-coding RNAs or lincRNAs, are lncRNAs that are located in between genes (160–165). As the investigation of the structure of lncRNAs is still in its early stages, we will provide a summary of the existing structural data on lncRNAs. Regarding the structure and composition of lncRNA, there are three fundamental levels: primary, secondary, and tertiary structures (164). Recent insights into the structural architecture of lncRNAs have enhanced our comprehension of the molecular mechanisms that facilitate the functioning of these RNAs. Furthermore, protein binding sites can be identified inside secondary structures by the presence of duplexes, bulges, hairpins, internal loops, and junctions. These structures serve as the fundamental framework of lncRNAs that control areas without pairing and perform Watson–Crick base pairing (166). In addition, the complex organization of lncRNAs provides sites for interactions and ensures the stability of lncRNAs through the formation of a triple helix at the 3′ end of the lncRNA. The triple helix is used to stabilize lncRNAs that lack a poly(A) tail (167). LncRNAs have a fascinating role in modulating proteins, genes, and ncRNAs. Furthermore, recent research has emphasized the disruption of lncRNAs in different types of human tumors and their connection to the fundamental characteristics and biological processes of cancer (168–170). **Figure 4** is an overview of miRNA regulation by lncRNAs in cancer.

LncRNAs demonstrate interaction with other molecular targets in human cancers. LncRNA LITATS1 has been shown to suppress EMT and enhances the degradation of TβRI to impair cancer cell plasticity (171). LncRNA MIR17HG is another factor that has been shown to suppress progression of breast cancer, and this factor sponges miR-454-3p to increase levels of FAM135A (172). Similar to miRNAs, the lncRNAs can be enriched in exosomes, and they are main regulators of miRNAs in cancer (173). The SLC31A1 has been considered as a regulator of cuproptosis in breast tumor, and LINC01614 exerts a modulatory impact on this gene (174). The high expression of HOTAIR in breast tumor mediates unfavorable prognosis, and it demonstrates interaction with miR-129-5p (175). Therefore, lncRNAs have an interaction with various molecular pathways in human cancers including ovarian tumor (176, 177).

### 3.2 LncRNAs in the regulation of proliferation and metastasis

Cancer cells exhibit uncontrolled proliferation in the absence of any external stimulants (161). This is a distinguishing feature exhibited by cancer cells. Cancer cells undergo alterations in the manufacture or function of chemicals that promote or inhibit growth, enabling them to bypass signals that regulate cell proliferation and attain unrestricted growth (89). Investigating the expression of lncRNAs has revealed the existence of lncRNAs that can either stimulate or hinder cell proliferation in many types of cancer, such as prostate cancer. The two lncRNAs that have received the most extensive research attention are HULC and LALR. Both of these lncRNAs have the capacity to impact cell growth by selectively interacting with many essential regulators in separate biological processes. Du and colleagues (178) found that HBV X protein (HBx) can increase the expression of HULC, leading to the promotion of hepatocyte proliferation by reducing the levels of p18 (hyphantria cunea nucleopolyhedrovirus). Their research has demonstrated that HBx can stimulate the HULC promoter via the CREB pathway. By suppressing HULC in a time-dependent manner, there was a significant decrease in cell proliferation in HBx stably transfected cell lines. Conversely, the excessive expression of HULC resulted in an augmentation of cell proliferation in L-O2 cells, which are derived from the parental HepG2 cells. This discovery is of great significance. The study demonstrated that the xenografts derived from same cells in nude mice exhibited equivalent results. Moreover, there is a belief that p18, a tumor suppressor, translocates to the nucleus to initiate the activation of the p53 pathway (179). It is also considered a target gene in human urothelial carcinoma. Subsequent examination uncovered that HULC suppresses the manifestation of p18 in both laboratory and living organism environments, thereby promoting the growth of liver cells. However, it is unclear from this article whether HULC downregulates p18 through direct binding or indirect interaction. Additional validation is necessary now. A further instance of an lncRNA that contributes to cell proliferation is the lncRNA-LALR1 (lncRNA associated with liver regeneration), which consists of approximately 480 bp in mice. According to Xu’s research, LALR1 is specifically increased in hepatocytes after two-thirds partial hepatectomy in mice. The overexpression of this factor can reduce the G0/G1 population in hepatocytes, resulting in an increase in cell proliferation. For a more in-depth examination of the procedure, it is important to mention that LALR1 can inhibit AXIN1 and, thus, stimulate the Wnt/β-catenin pathway in mouse hepatocytes. More specifically, LALR1 can interact with the transcription factor CTCF, causing CTCF to bind to the AXIN1 promoter region and inhibit the production of the gene. The stability of the β-catenin destruction complex decreases when the expression of Axin1 decreases. Meanwhile, proteins like T-cell factor-4 and lymphoid enhancer factor attach to active β-catenin. After being moved to the nucleus, it has an impact on the transcription of particular genes, such as c-myc and cyclin D1. LALR1 regulates the progression of the mouse cell cycle and promotes the growth of hepatocytes. Importantly, research has demonstrated that human liver tissues express a human ortholog of LALR1 (hLALR1), which is a significant discovery. Further investigation is necessary to ascertain the extent to which it exhibits similarities to mouse LALR1 (180).

A growing corpus of studies has demonstrated that the primary tumor is not the primary cause of mortality for many individuals; instead, the metastases are responsible for their demise. The initial stage of both invasion and metastasis involves the process of intravasation, where the cancer cells enter the nearby blood and lymphatic vessels. The following stages involve micrometastases, extravasation, transit through the lymphatic and hematogenous systems, colonization, and intravasation (89, 181). Recent research has demonstrated that a substantial quantity of lncRNAs associated with HCC have important functions in the cellular processes of invasion and metastasis. LncRNA-Dreh, a prototypical example, is an lncRNA that experiences downregulation in expression due to the presence of HBx. It was found that the level of Dreh expression was significantly decreased in HBx-transgenic animals and in mouse liver cells that expressed HBx. Both in laboratory experiments (*in vitro*) and in living organisms (*in vivo*), it has been observed that reducing the activity of cellular Dreh is linked to an elevation in the ability of liver cancer cells to migrate and invade surrounding tissues. Studies have demonstrated that Dreh has a higher affinity for vimentin compared to other molecules. Vimentin is the primary structural element of the cytoskeleton in mesenchymal cells and belongs to the type III intermediate filament family. The prevention of HCC metastasis is achieved by altering and reorganizing the expression of vimentin. Transfected cells expressing Dreh exhibit a significant decrease in vimentin levels, accompanied by the development of helical filaments that span from the nuclear membrane to the cellular membrane. However, when subjected to regulation, filament formations withdraw toward the nuclear membrane of the cells. Furthermore, the cytoplasm of cells transfected with Dreh exhibits a substantial presence of filamentous aggregation, together with a considerable quantity of irregular fragmented aggregated structures. Owing to the many alterations made to vimentin, the cells would experience instability, resulting in the facilitation of cell migration. Additionally, a human counterpart of Dreh (hDREH) has frequently been observed to be downregulated in tissues affected by HBV-related HCC. This drop in expression has been strongly associated with lower survival rates among patients with HCC. Given these information, it is crucial to prioritize further investigation of the hDREH in order to further therapeutic approaches (182). Another lncRNA called LET, which stands for low expression in tumor, has been found to have a substantial role in hypoxia-induced metastasis in HCC. LET levels are commonly reduced in various types of cancer, such as squamous cell lung carcinoma, colorectal cancer, and HCC. Through the use of orthotopic tumor models in nude mice, experiments involving both the enhancement and suppression of LET activity have shown that LET effectively reduces the infiltration of the liver and the dissemination of metastases to the abdominal area. Ultimately, Yang and colleagues (183) successfully discovered a pathway that includes hypoxia-inducible factor 1, alpha subunit (HIF-1a), histone deacetylase 3 (HDAC3), and leaflet/NF90. In hypoxic settings, HDAC3, which is controlled by HIF-1a, can inhibit LET via decreasing the histone acetylation-dependent control of the LET promoter region. LET downregulation leads to a decrease in direct interactions between LET and NF90. Consequently, this results in an improvement in the stability of NF90, finally causing a rise in the synthesis of HIF-1a. HIF-1a is a specific mRNA that NF90 targets and is involved in the process of metastasis induced by hypoxia. Thus, the spread of HCC is inhibited by LET through this mechanism of positive feedback. The presence of this feedback loop considerably increases the complexity of the gene regulation network.

The lncRNAs demonstrate interaction with various molecular targets in HCC (184). The lncRNA GBAP1 is stimulated by METTL3, and through the induction of the BMP/SMAD axis, it is able to enhance tumorigenesis (185). The lncRNA SNHG20 has been shown to downregulate miR-5095 and upregulate MBD1 in HCC progression (186). LncRNA SATB2-AS1 is another factor capable of impairing HCC development through suppression of STAT3/HIF-1α and enhancing GRIM-19 levels (187). The lncRNA SNHG1 has the tumor-promoting function in HCC and it can downregulate miR-199a-5p/3p to enhance levels of FANCD2 and G6PD in reducing ferroptosis (188). A number of lncRNAs respond to the hypoxia in the tumor microenvironment. The hypoxia-mediated increase in the levels of lncRNA MRVI10AS1 can recruit the CELF2 as an RNA-binding protein (RBP) to enhance SKA1 stability in increasing HCC progression (189). The cuproptosis-associated lncRNAs can be considered as prognostic factors in HCC (190). The lncRNA HClnc1 is able to induce PKM2 expression and mediates poor survival rate in HCC (191). The lncRNA SLC7A11-AS1 increases the ubiquitination of KLF9 to enhance tumorigenesis in HCC (192). The lncRNA PTOV1-AS1 overexpression modulates miR-505 expression, and it is able to stimulate sorafenib resistance in HCC (193). Taking everything together, the current studies highlight that lncRNAs are versatile factors in HCC and can modulate various hallmarks with different downstream targets (194–196).

### 3.3 LncRNAs in the regulation of drug resistance in HCC

Considerable progress has been made in the treatment of HCC, thanks to the development of medications. Various anticancer therapies have been employed in this regard. Multiple investigations have been undertaken to investigate the development of treatment resistance in HCC (197–200). Zhang and colleagues (201) discovered that the prevention of miR-15a-5p in HCC leads to resistance to therapy due to the enhanced expression of elF4E. Insufficient radiofrequency ablation in HCC can lead to treatment resistance due to an increase in the expression rate of Hsp70. The suppression of miR-15a-5p in HCC leads to treatment resistance through the overexpression of elF4E. As a result of an increase in the expression level of Hsp70, it has been observed that insufficient radiofrequency ablation can lead to treatment resistance in HCC (202). EZH2 in HCC downregulates the expression of miR-381, resulting in the activation of the Akt signaling pathway, which is accountable for the development of treatment resistance (203). This section discusses the role of lncRNAs in HCC treatment resistance regulation. There are two principal types of lncRNAs in this context: those that operate as chemosensitizers and those that can create drug resistance. That is why it may be possible to elevate HCC cell susceptibility to drugs by hindering carcinogenic lncRNAs. Silencing LINC00173 lowers resistance to cisplatin and is related to a poor outcome in HCC. LINC00173 downregulates miR-641, which, in turn, regulates RAB14 expression, causing cisplatin resistance in HCC (204). Other research looked at how anticancer medications influenced HCC cell survival levels and discovered that these agents instigate cell death (205, 206). Consequently, inhibiting apoptosis in tumor cells might lead to the development of drug resistance, a common trait observed in HCC. ST8SIA6-AS1, a lengthy non-coding RNA, has been demonstrated to hinder apoptosis and expedite the proliferation of HCC cells. The cytoplasmic lncRNA ST8SIA6-AS1 functions as a mediator for the sequestration of miR-4656, resulting in the upregulation of HDAC11 expression and the consequent inhibition of apoptosis in HCC (207). Hence, in order to make HCC cells more responsive to treatment, apoptosis is generally advised.

Growing evidence proposes that the lncRNA NEAT1 has a prominent role in the development of treatment resistance in HCC. During the development of sorafenib resistance in HCC, the lncRNA NEAT1 triggers the activation of protective autophagy. NEAT1 is a lengthy RNA molecule that functions as a mediator for miR-204 sponge, which increases the expression of ATG3 and ultimately triggers autophagy (208). A recent study has demonstrated the involvement of autophagy in the development of treatment resistance in HCC. According to the findings of this study, LINC00160 acts as a decoy by reducing the expression of miR-132, leading to an increase in ATG5 expression. Additionally, it enhances autophagy and promotes treatment resistance in HCC via reducing the expression of p62 (209). The lncRNA NEAT1 is observed to have increased expression in HCC. Considering this, it should have oncogenic activity. The study conducted by Niu and colleagues (210) uncovered that NEAT1 is the causative factor in stimulating the production of miR-149-5p. This, in turn, activates Akt1 signaling and finally leads to the development of resistance to sorafenib in HCC. Increased expression of BLCAF1 has been correlated with enhanced proliferation and invasion of HCC cells, and it plays a considerable role in this process. BLCAF1 is responsible for enhancing NEAT1 expression, which actuates chemoresistance in HCC (211). It can be inferred that suppressing the lncRNA NEAT1 expands the drug sensitivity of HCC cells. The lncRNA/STAT3 axis is involved in modulating the drug susceptibility of HCC cells. An observation has been made that the lncRNA DANCR is excessively produced in HCC. Furthermore, it has been demonstrated that the suppression of this gene is related to the vulnerability of tumor cells to sorafenib. Liu and colleagues’ research (212) reveals that the lncRNA DANCR enhances STAT3 signaling, leading to the development of sorafenib resistance in HCC. Several lncRNAs have represented dual functions as both oncogenes and onco-suppressors in HCC. This specific occurrence is observed in the lncRNA H19 in HCC. Increasing evidence indicates that the lncRNA H19 plays a function in promoting cancer development in HCC. In a study conducted by Wu and colleagues (213), it was discovered that polymorphisms of the lncRNA H19 may increase the risk of developing and beginning HCC.

### 3.4 Therapeutic targeting of lncRNAs in hepatocellular carcinoma

Presently, the outlook for HCC is often unfavorable, mostly due to the lack of a specific treatment target. Sorafenib, which specifically targets receptor tyrosine kinases (RTKs), is the most commonly prescribed targeted medicine for the treatment of HCC. Resistance to sorafenib is commonly observed in the treatment of HCC (214, 215). In summary, antisense oligonucleotides (ASOs) are specific lncRNAs that bind to short single-stranded DNAs, resulting in the formation of a DNA–RNA complex. RNase H has the ability to identify and break down this particular combination. On the other hand, RISCs are formed when short double-stranded RNAs are attached to the AGO2 protein. Subsequently, it is necessary for them to associate with specific lncRNAs in order to create an RNA–RNA complex, which will subsequently facilitate the suppression of lncRNAs (216). Considering these differences, it is probable that ASOs and RNAi demonstrate varying degrees of effectiveness in silencing, depending on several factors, such as the specific location inside the cell where the targeted lncRNAs are found. Within nuclei, ASOs have demonstrated more efficacy compared to RNA interference (RNAi). However, RNAi has proven to be more effective than ASOs in targeting cytoplasmic lncRNAs (217). This could be due to the fact that RNase H is predominantly located in the nucleus, while RISC largely carries out its duties in the cytoplasm (218, 219). Delivery of tumor-suppressive lncRNAs could potentially serve as an alternative method to knock down oncogenic lncRNAs by delivery. PRAL, for example, is an lncRNA that acts as a tumor suppressor by stabilizing p53. Furthermore, it has been shown that the administration of PRAL by an adenovirus vector may noticeably lessen the growth of HCC in tumor-bearing mice, indicating that it may have potential clinical applications for the treatment of HCC (220). It is important to note that there are a significant number of HCC-targeted ASOs and RNAi therapies available, as they have already been used to treat HBV. In summary, ASOs and siRNAs undergo modifications through chemical conjugates [such as N-acetylgalactosamine (GalNAc)] (221) or are encapsulated in delivery vehicles (such as lipid nanoparticles) (222), leading to an enhanced pharmacokinetic profile. The successful application of ASOs and RNAi in treating HBV establishes a strong foundation for the treatment of HCC and lncRNAi.

### 3.5 LncRNAs as ceRNA of miRNAs in hepatocellular carcinoma

Furthermore, lncRNAs has the capacity to engage in competition with endogenous RNA and act as “sponges” for miRNAs, thereby regulating the synthesis of target mRNAs. Recent research indicates that several lncRNAs linked to HCC possess the capability to regulate the expression of specific genes by binding to miRNAs (223). This hinders the binding of specific miRNAs to their target mRNAs. The miR-200 family is responsible for inhibiting the process of EMT and the spread of tumors to other parts of the body (224). The human chromosome 11p15.5 serves as the origin of transcription for H19, an lncRNA that is evidently suppressed in HCC. H19 possesses the capability to attach itself to hnRNPU/PCAF/RNAPol II, leading to an augmentation in histone acetylation and the initiation of miRNA-200. The latter hinders the proliferation of malignancies and counteracts the consequences of the EMT (225). In summary, ASOs are specific lncRNAs that bind to short single-stranded DNAs, resulting in the formation of a DNA–RNA complex. RNase H can identify and break down this particular combination. On the other hand, RISCs are formed by attaching short double-stranded RNAs to the AGO2 protein. Subsequently, they must associate with certain lncRNAs to create an RNA–RNA complex, which will subsequently facilitate the suppression of lncRNAs (226). Vertebrate H19 contains both canonical and non-canonical binding sites for let-7. Let-7 is a pivotal protein involved in the pathogenesis of HCC. H19 functions as an *in vivo* molecular sponge and conducts genome-wide transcriptome profiling. It plays a role in controlling the availability of let-7 (227). It is clear that lncRNAs reduce the regulation of miRNAs by functioning as miRNA sponges. Moreover, miRNAs possess the capacity to directly engage with lncRNAs and inhibit their synthesis (228, 229). The HOXA distal transcript antisense RNA (HOTTIP) is a non-coding antisense transcript that is situated at the farthest end of the HOXA gene cluster. It has the ability to regulate the expression of HOXA genes in close proximity to HCC. An important function of miR-125b in liver cancer is to control the activity of HOTTIP following the completion of transcription. There is compelling evidence indicating a clear link between HOTTIP and miR-125b. It is commonly seen that when miR-125b is downregulated, there is often an increase in HOTTIP expression (230). In the context of liver cancer, miR-192 and miR-204 act as tumor suppressors. Although argonaute 2 (Ago2) is predominantly located in the cytoplasm, additional RNAi factors, including Dicer, TRBP, and TRNC6A/GW182, are also found in the nucleus of the cell. These proteins play a crucial role in the regulation of functional RNAi (231). HOTTIP can partake in physical interaction with Ago2. One notable attribute exhibited by cancer cells is the presence of glutaminolysis (GLS1). As the enzyme that catalyzes the transformation of glutamine to glutamate, mitochondrial GLS1 is significantly critical to the glutaminolysis process (232). Both miR-204 and miR-192 can decrease HOTTIP expression via the Ago2-mediated RNAi pathway. The next step is to silence GLS1 expression in HCC cells, which leads to a drastic decrease in HCC cell viability (233).

### 3.6 Clinical importance, diagnosis, and prognosis

**Figure 5:**
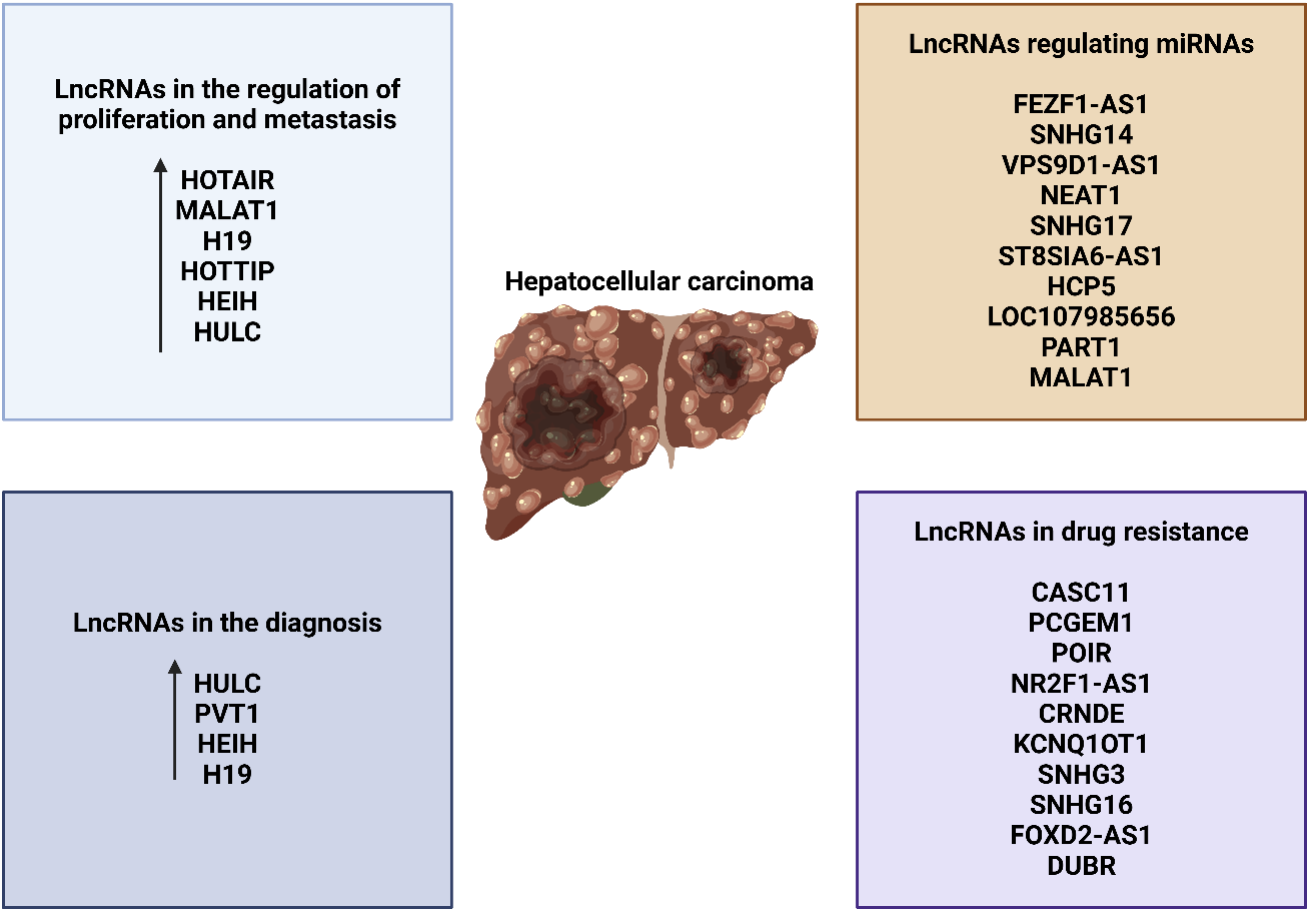
The function of lncRNAs in diagnosis, therapy resistance, and progression.

The association between several lncRNAs and clinicopathologic characteristics and prognosis in several types of cancers (234–238) highlights the potential of lncRNAs as biomarkers for determining prognosis. Out of the 74 lncRNAs that are not functioning properly in HCC, 63 are linked to the clinicopathologic characteristics of the illness. These characteristics include size, focality, differentiation, encapsulation, invasion, metastasis, disease stage, survival, nontumor traits (such as cirrhosis), serum alpha-fetoprotein (AFP), and the presence or absence of HBV infection. Specifically, it was shown that the majority of lncRNAs were linked to factors such as overall survival, cancer stage, and tumor size. This result enhances the importance of lncRNAs as indicators for prognosis. The rationale for this is that variables such as tumor size, cancer stage, and patient survival rate have a substantial influence on the probability of survival. It is crucial to note that several lncRNAs are strongly linked to many clinicopathologic variables. Cirrhosis, tumor invasiveness, blood AFP levels, HBV status, metastatic disease, HCC stage, and survival are all attributes encompassed within this classification. Six specific lncRNAs—PANDAR, MALAT-1, GPC3-AS1, CARLo-5, UCA1, and LOC90784—have been found to have a substantial correlation with six to eight distinct clinicopathologic characteristics. These findings indicate that these six lncRNAs have the capacity to establish a characteristic of a negative prognosis. Similarly, three lncRNAs (FTX, AOC4P, and linc-p21) that have been reduced in expression have been discovered to be significantly linked to six different clinicopathologic characteristics. Consequently, they have the potential to function as a distinctive indicator of a favorable prognosis. It is important to note that among the lncRNAs that showed either increased or decreased levels, a total of 17 were shown to be clinically associated with distinct tumor characteristics, such as size, location, degree of differentiation, invasion, and spread to other parts of the body. The efficacy of these lncRNAs in modulating cancer-related cellular markers has been confirmed by experiments conducted utilizing *in vitro* and *in vivo* mouse models. Cancerous growth is distinguished by specific characteristics, including tumor size, cell cycle, apoptosis, cell transformation, cell migration, invasion, and metastasis. Out of the total of 16 increased lncRNAs, 6 have been proven to play significant roles in the growth of tumors and the spread of metastasis. These six RNAs are GLTC, PCAT-14, SNHG12, MALAT-1, Linc00462, and PDIA3P1. It has been reported that these six lncRNAs modulate key cancer pathways, such as Hedgehog, NF-κB, FGFR1/ERK, mTOR, PI3K/AKT, and p53. On one hand, the downregulation of linc-p21 activates the Notch signaling pathway. On the other hand, the downregulation of uc.134 deactivates the Hippo kinase pathway. The deregulation of these eight lncRNAs, which target crucial cancer pathways and have been validated both clinically and experimentally, showed substantial correlations with tumor characteristics, vascular invasion, metastasis, cancer stage, and survival. This finding reaffirms the potential of these lncRNAs as potential prognostic biomarkers and for the development of drugs (**Figure 5**) (239). The lncRNAs WRAP53 and UCA-1 are also diagnostic factors in HCC (NCT05088811). However, more clinical studies are required to understand the role of lncRNAs in HCC.

## 4 CircRNAs in hepatocellular carcinoma

### 4.1 Biogenesis of circRNAs and their interaction with molecular pathways

**Figure 6:**
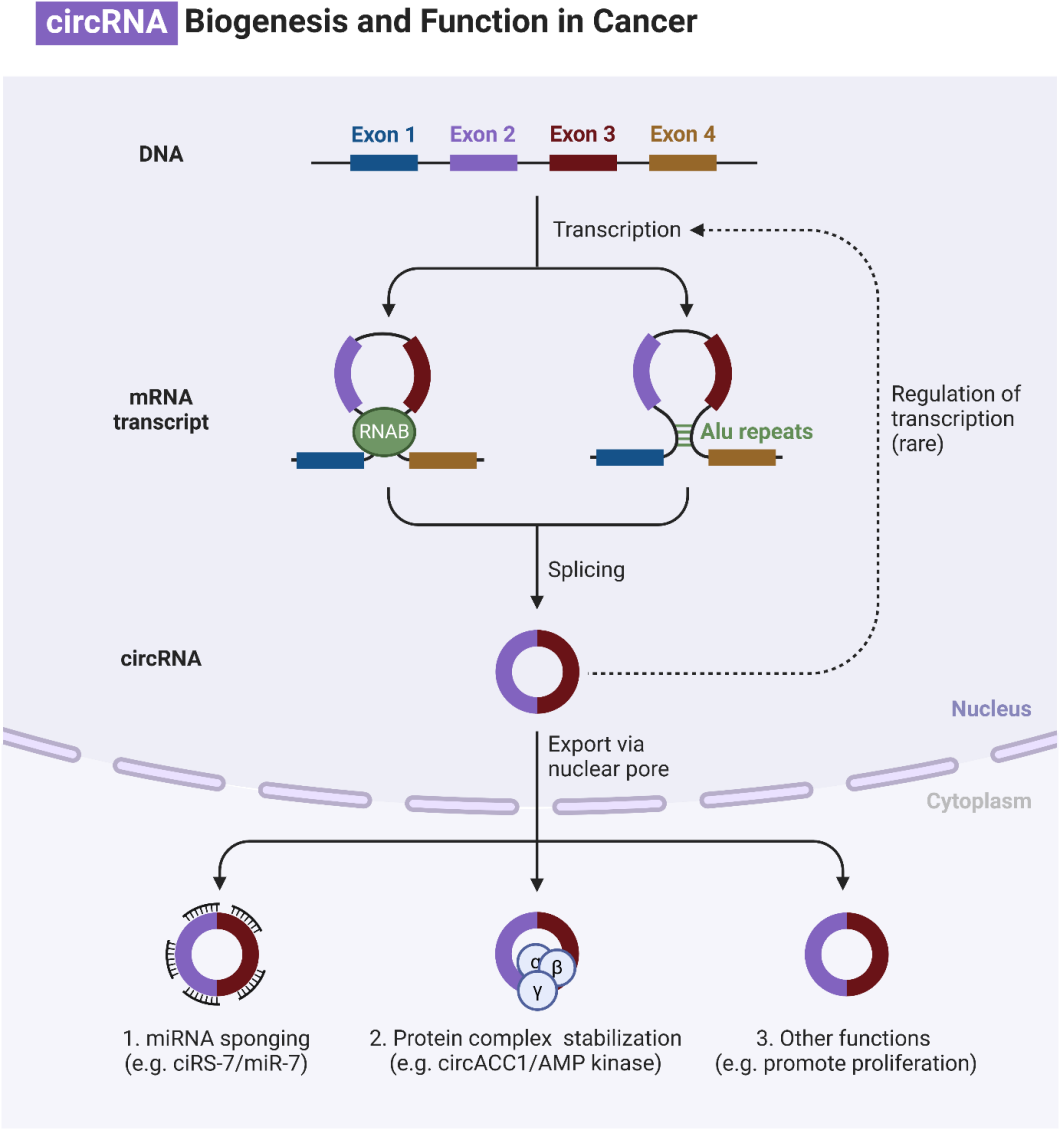
An overview of circRNAs in cancer.

Circular RNAs (circRNAs) are generated through lariat-circulating or back-splicing mechanisms, originating from various regions of the genome (240). Exons are the predominant origin of circRNAs, although intergenic, intronic, antisense, and UTR sections can also serve as potential sources, albeit extremely rarely. The designations circRNAs, EIciRNAs, and ciRNAs are used in the order of their origin, specifically from the exon, exon and intron, and intron, respectively. The majority of circRNAs are predominantly localized in the cytoplasm, although they can also be found in other subcellular compartments (241–244). The production of circRNA is affected in many ways by various sequence characteristics. Lengths of introns and exons, repetitive sequences, and RBPs are all examples of such factors. One type of RBP is the quaking protein, another is the heterogeneous nuclear ribonucleoprotein L, and yet another is the muscleblind protein (245–250). Liang and colleagues (251) have recently found, by the use of RNAi screening, that the proportion of linear to circRNA expression is regulated by numerous core spliceosomal and transcription termination factors. How are circRNAs degraded or eliminated? Lately, researchers have been focusing their efforts on exosomes. Exosomes are vesicles with two layers of membranes that contain various functional molecules, such as proteins, miRNAs, lncRNAs, and circRNAs. They have the ability to enhance cell-to-cell communication through either paracrine or endocrine mechanisms (252). CircRNAs can potentially undergo clearance or degradation through exosomal release, a mechanism that involves the removal of circRNAs from the plasma membrane (253). **Figure 6** is an overview of circRNAs in cancer.

Understanding the interaction of circRNAs with downstream targets in human cancers can highlight the underlying mechanisms involved in carcinogenesis. In breast tumor, circ_0001667 is able to downregulate miR-6838-5p in increasing CXCL10 expression to accelerate tumorigenesis and mediate angiogenesis (254). Hsa_circ_0063329 is another factor capable of miR-605-5p suppression to increase TGIF2 expression in prostate cancer suppression (255). The circ-hnRNPU is a regulator of tumorigenesis and glycosylation that can suppress NONO-induced c-Myc transactivation (256). circRNA hsa_circ_0067842 has been shown as a regulator of tumorigenesis in breast cancer that enhances invasion and induces immune evasion through PD-L1 upregulation (257). In most cases, the action of circRNAs is related to the modulation of miRNA expression through sponging (258–262). Hsa_circ_0001278 inhibits miR-338-5p to increase AMOTL1 to enhance colorectal cancer progression (263).

### 4.2 CircRNAs exert dual function in hepatocellular carcinoma

The fact that circRNAs are made in specific tissues makes it likely that they contribute to the development of various diseases (264–267). The consequences revealed that several malignancies, including HCC, exhibit an upregulation of the cancer-causing circRNA known as CDR1as (268). Previous research found that elevated CDR1as levels in HCC tissues were highly associated with invasion of the liver microvasculature and only weakly with HCC development (269). Scientists demonstrated that HCC cell growth and invasion were reduced when CDR1as was inhibited. Recent studies have shown that CDR1as binds to miR-7 and acts as a sponge. HCC cell growth and invasion were inhibited by increasing miRNA-7 expression. Both cyclin E1 (CCNE1) and phosphoinositide 3-kinase catalytic subunit δ (PIK3CD), which are their target genes, saw a decrease in transcription. The PIK3CD/phospho-p70 S6 kinase (p70S6K)/mammalian target of rapamycin (mTOR) signaling pathway is disrupted when CDR1as functions as a miR-7 sponge, enhancing the proliferation and invasion of HCC cells. These results point to a critical function for CDR1as in controlling HCC progression. Furthermore, CDR1as-regulated proteins in HCC cells were identified using a quantitative proteomics approach (270). After conducting the proteomic analysis and functional verification, it was demonstrated that the overexpression of CDR1as has the capacity to enhance the proliferation and progression of the cell cycle in HCC cells. One way to achieve this is by controlling the signaling of epidermal growth factor receptor (EGFR) through the modulation of miR-7 expression. Tumor cells undergo a process called EMT to become metastatic and invasive. During EMT, the tumor cells lose E-cadherin and gain vimentin (271–274). Recent findings have represented that the transcription factor Twist1, known for its ability to trigger EMT, plays a role in upregulating the expression of Cul2 circRNA (circ-10720) (275). Circ-10720 exhibited a significant connection with the occurrence of malignant tumors and a worse prognosis in HCC. Upon conducting additional analysis, it was revealed that circ-10720 promoted the growth, movement, and infiltration of HCC cells. Twist1 may increase the expression of vimentin by increasing the levels of circ-10720, which binds to a group of miRNAs that specifically target vimentin. This implies that the procedure involved in this can be delineated as follows. Therefore, it was discovered that the Twist/circ-10720 pathway has a beneficial impact on the progression of EMT in lung cancer. However, the tumor-promoting function of Twist1 was abolished both in laboratory experiments (*in vitro*) and in living organisms (*in vivo*) when circ-10720 was employed to suppress it. The results indicated that circ-10720 plays a role in promoting the development of HCC and suggests that circ-10720 could be a potential target for therapeutic intervention in HCC treatment. This analysis also uncovered some distinctive perspectives on therapy approaches for HCC intervention that are founded on circRNA. The development of cancer and its progression are greatly impacted by aquaporin 3, sometimes referred to as AQP3 (276). Scientists demonstrated an association between high AQP3 expression levels and tumor growth, metastasis, and prognosis of patients with HCC (277, 278). AQP3 levels were markedly increased in the HCC tissues where it was identified. A study revealed that miR-124-3p is noticeably suppressed in HCC and hinders the proliferation and migration of HCC cells by targeting AQP3 (279). Additional study has revealed that circHIPK3 functions as a sponge for miR-124-3p, hence regulating the production of AQP3. CircHIPK3 has the ability to enhance the growth and movement of HCC cells by interacting with the miR-124-3p/AQP3 axis. An *in vivo* study showed that suppressing circHIPK3 led to a reduction in the progression of HCC. Furthermore, the role of another circRNA known as hsa_circ_0000673 in the progression of HCC has been revealed (280). According to the data, hsa_circ_0000673 was shown to be highly expressed in HCC tissues. Knocking down hsa_circ_0000673 led to a significant reduction in the growth and spread of HCC cells, as well as a prevention of tumor formation in living organisms. Hsa_circ_0000673 functioned as a miR-767-3p sponge, leading to an upregulation of its downstream effector SET. This was achieved through a mechanistic process. Moreover, there is a correlation between the atypical expression of SET and the proliferation of cancer (281, 282). SET has been found to act as an oncogene during the development of cancer. Not only was it found that the levels of SET were increased in HCC, but it was also established that there is a strong correlation between high SET levels and unfavorable clinical outcomes (283). Hsa_circ_0000673 played a role in promoting HCC malignancy via influencing the miR-767-3p/SET pathway.

There was a notable disparity in the expression of circRNA SMAD2 (circSMAD2) between HCC tissues and the adjacent normal tissues (284). A significant association was seen between CircSMAD2 and the extent of differentiation observed in HCC tissues. Overexpression of circSMAD2 has the capacity to inhibit the migratory, invasion, and EMT responses of HCC cells. This study has verified that miR-629 is the specific target of circSMAD2. Moreover, miR-629 has the capacity to mitigate the impact of circSMAD2 on the progression of HCC. Furthermore, it was found that the expression of circC3P1, a different circRNA, was reduced in HCC (285). Overexpressing CircC3P1 significantly suppresses the proliferation, migration, and invasion of colon cancer cells. Moreover, circC3P1 suppressed the progression of HCC and its metastatic potential in live organisms. An important finding is that circC3P1, by acting as a sponge for miR-4641 in HCC cells, resulted in an upregulation of phosphoenolpyruvate carboxykinase 1 (PCK1) expression. The considerable reduction in the proliferation, migration, and invasion of HCC cells was seen when the expression of PCK1 was inhibited by circC3P1. By specifically targeting miR-4641 in HCC, circC3P1 successfully fulfills its role as a tumor suppressor. This was achieved by upregulating the expression of PCK1. Further findings indicated that hsa_circ_0005986 functioned as a tumor suppressor in the progression of HCC (286). The expression of hsa_circ_0005986 was significantly downregulated in HCC tissues compared to healthy tissues. The downregulation of hsa_circ_0005986 led to the release of miR-129-5p, resulting in a decrease in the expression of its target gene, Notch1. The downregulation of hsa_circ_0005986 had a significant impact on the cell cycle transition, leading to an increase in the proliferation of HCC cells. Moreover, there was a direct relationship between the decreased expression level of hsa_circ_0005986 and the clinicopathological characteristics of patients with HCC. These attributes encompassed the dimensions of the tumor, the presence of microvascular invasion (MVI), and the stage of liver cancer as assessed by the Barcelona Clinic Liver Cancer (BCLC) classification. Thus, hsa_circ_0005986 has the potential to not only slow the process of HCC carcinogenesis, but also serve as a possible biomarker for diagnosing HCC. Yu and colleagues (287) utilized RNA-sequencing technology to compare the expression of circRNAs in paired HCC and surrounding non-tumorous tissues from two groups. The cSMARCA5 (hsa_circ_0001445) circRNA has been accurately characterized and originates from exons 15 and 16 of the SMARCA5 gene. The role of cSMARCA5 in the progression of HCC was investigated. The results indicated that the expression of cSMARCA5 was reduced in many HCC tissues. Patients with HCC who underwent surgical removal of the tumor showed a decrease in the expression of cSMARCA5, which was associated with more aggressive clinicopathological characteristics. This indicates that it could serve as a risk factor for both overall survival and recurrence-free survival in those individuals. Research indicates that the overexpression of cSMARCA5 can impede the migration and proliferation of HCC cells. cSMARCA5 increased the expression of the widely recognized tumor suppressor tissue inhibitor of metalloproteinase 3 (TIMP3) by acting as a sponge for miR-17-3p and miR-181b-5p. These findings not only provide new insights into the role of circRNAs in HCC development, but also highlight the importance of cSMARCA5 in promoting HCC growth and metastasis. A separate study (288) discovered that the presence of hsa_circ_0001445 was observed in both HCC and pericancerous tissues that were appropriately paired. The findings revealed a significant decrease in the expression of hsa_circ_0001445 in HCC tissues. This decrease showed an inverse correlation with the number of tumor foci. Based on gain-of-function investigations, overexpression of hsa_circ_0001445 can lead to cell death and limit the ability of HCC cells to move, invade, and multiply in a laboratory setting. The results indicate that hsa_circ_0001445 plays a regulatory role in the progression of HCC.

The circRNAs show interaction with other pathways in HCC. The circ_0002003 overexpression can enhance tumorigenesis through sponging miR-1343-3p to increase DTYMK, DAP3, and STMN1 levels (289). As an anticancer compound, sevoflurance has been shown to upregulate circ_0001649 to upregulate SGTB through miR-19a-3p inhibition in impairing HCC progression (290). Therefore, anticancer compounds can regulate circRNAs in HCC (291, 292). Circ-MBNL3 suppresses miR-873-5p expression to enhance PHF2 levels in impairing HCC progression (293). Hsa_circ_0093335 inhibits miR-338-5p expression to elevate HCC malignancy (294). Hsa_circ_0119412 is another factor that suppresses miR-526b-5p in enhancing STMN1 levels to facilitate tumorigenesis (295). Circ_0124208 downregulates miR-338-3p to upregulate LAMC1 and can be used as a biomarker in HCC (296). Circ_0082319 undergoes upregulation by HuR, and it can sponge miR-505-3p to upregulate PTK2 in tumorigenesis induction (297).

### 4.3 CircRNAs in the regulation of therapy resistance and therapeutic perspective

Resistance to therapy is a major factor in the reappearance and spread of HCC, and it poses a considerable obstacle to tumor treatment (298, 299). Recently, several studies have found that specific dysregulated circRNAs play a role in the resistance of HCC to chemotherapy and radiation, respectively. CircRBXO11 promotes tumor growth and confers resistance to oxaliplatin in HCC cells by sponging miR-605, which targets FOXO3, and activating ABCB1 (300). Moreover, studies have shown that circ_0003418 functions by suppressing the development of cancer and resistance to cisplatin chemotherapy in HCC through the Wnt/β-catenin pathway (301). Furthermore, circRNA 101505 increases NOR1 expression and absorbs miR-103, making HCC cells less sensitive to cisplatin (302). In addition, cZNF292 has the ability to increase the radiosensitivity of hypoxic HCC cells by decreasing the Wnt/β-catenin pathway and effectively enhancing the nuclear translocation of SOX9 (303). Moreover, Wu and colleagues found that the circRNAs’ profile of HCC could potentially serve as biomarkers for individuals with sorafenib-resistant HCC (304). Sorafenib resistance in HCC is sustained by two primary pathways, according to Xu and colleagues. During the first step, the Wnt/β-catenin pathway is launched and miR-103a-2-5p/miR-660-3p is active. Since the N6-methyladenosine modification increased RNA stability, it was found to improve the expression of circRNA-SORE (305). In addition, circRNA-SORE formed a connection with and stabilized YBX1 by inhibiting PRP19-mediated YBX1 degradation. In addition, HCC cells demonstrated the ability to develop resistance to sorafenib by means of the translocation of circular RNA-SORE through exosomes (306).

The progression of HCC is closely linked to specific circRNAs that show increased expression. Therefore, circRNAs have the potential to be targeted therapeutically for HCC. HCC cells, as well as individuals who have experienced HCC metastasis or recurrence, have demonstrated a heightened level of circASAP1 expression. Studies have shown that circASAP1 promotes the growth of HCC cells, as well as the formation of clusters, movement, infiltration, tumor growth, and spread to the lungs, both in laboratory settings and in living organisms. CircASAP1 has been shown to promote metastasis of HCC through the miR-326/miR-532-5p-MAPK1/CSF-1 signaling pathway. Hence, circASAP1 exhibits the capacity to function as a promising therapeutic target for HCC (307). The heightened expression of circTMEM45A in serum exosomes obtained from patients with HCC has the potential to be utilized as a new diagnostic and therapeutic target for patients with HCC (308). Furthermore, the presence of a regulatory network composed of circ-CDYL-centric ncRNAs, in conjunction with HDGF and HIF1AN, holds promise as valuable biomarkers and targets for the timely identification and management of HCC (309). Therefore, it is believed that increased circRNA expression in HCC aids in the development of cancer, whereas their suppression has the opposite impact in HCC. One way to limit the development of HCC and have an anticancer impact is to use short interfering RNAs that are developed to target the backsplicing junction sites of oncogenic circRNAs (310, 311). Some circRNAs that have their expression downregulated are potentially potential therapeutic targets because of their capacity to restrict tumor growth and prevent the formation of HCC. As an example, cSMARCA5 expression is significantly downregulated in HCC tissues, it is associated with growth and metastasis, and it may serve as a separate prognostic marker for patients with HCC even after hepatectomy. Both *in vivo* and *in vitro* investigations have demonstrated that cSMARCA5 inhibits the growth and spread of HCC. This suggests that cSMARCA5 has potential as both a diagnostic and a therapeutic tool for HCC (287). Han and co-workers discovered that circMTO1 expression was associated with a poor outcome for breast cancer patients. Reduced circMTO1 expression intratumorally accelerated HCC development *in vivo*, suggesting its utility for HCC-targeted therapy. The circMTO1 gene may one day be used as a diagnostic tool and treatment target for people afflicted with HCC (312). Moreover, Zhang and colleagues uncovered that the expression of circDLC1 was reduced in HCC, and this was directly linked to the prognosis of patients with HCC. The overexpression of circDLC1 was observed to restrict the proliferation and metastasis of lung cancer cells in both laboratory and living organism environments. CircDLC1 has the potential to function as both a target for therapy and a biomarker for predicting outcomes in persons with HCC (313). Despite the decrease in the expression of these circRNAs, it is plausible that inducing their overexpression in HCC cells or tissues via transfection could lead to substantial anticancer effects.

### 4.4 CircRNAs as ceRNA for miRNAs in hepatocellular carcinoma

Studies on the composition of circRNAs have shown that these RNA molecules possess a noticeable abundance of miRNA binding sites, which extend the interaction between circRNA and miRNA (265). Subsequent investigations have illustrated that circRNAs had the capacity to regulate the expression of genes from which they originate through their interaction with miRNA molecules (314, 315). An instance of this can be seen in the correlation between the excessive expression of has_circ_0005075 and the dimensions of the HCC tumor. This discovery revealed that has_circ_0005075 might be responsible for the proliferation of the tumor, and it indicated a remarkable diagnostic capacity (AUROC = 0.94). Moreover, by the use of GO and route analysis, it was anticipated that has_circ_0005075 will be involved in cell adhesion. This pathway exhibited strong associations with cell proliferation, invasion, and metastasis in people diagnosed with HCC. It was postulated that the biological activities of has_circ_0005075 may modulate some components of the tumor cell membrane through biological functions. On top of that, it was found that the gene has_circ_0005075 has the ability to interact with four different miRNAs, namely, hsa-miR-23b-5p, hsa-miR-93-3p, hsa-miR-581, and hsa-miR-23a-5p, resulting in a decrease in their production and functionality. The results demonstrated above indicate that a considerable level of has_circ_0005075 expression in HCC is associated with the development of tumors, suggesting that this gene could serve as a promising biomarker for HCC (316).

Another study discovered that HCC was associated with extremely high rates of the famous circRNA Cdr1as, also known as ciRS7 (317). This circRNA contained a precise match for miR-671 and more than 70 conserved binding sites for miR-7. Blocking Cdr1as can potentially impede the proliferation and spread of hair cancer cells. Furthermore, the expression of miR-7 was decreased throughout this period. Overexpression of miR-7 can potentially suppress the expression of CCNE1 and PIK3CD, two genes that it specifically targets. Suppressing Cdr1as led to a reduction in the expression of several genes. These findings suggest that Cdr1as, apart from its role in targeting miR-7 in HCC, may also function as an oncogene (317). A research conducted by Xu and colleagues showed that the expression of Cdr1as was decreased in HCC. In addition, the study identified a strong association among the expression of Cdr1as and several factors: age below 40 years, serum AFP levels of 400 ng/μL or higher, hepatic MVI, and two specific genes targeted by miR-7, namely, PIK3CD and p70S6K (269). A similar discovery was made, indicating that the presence of Cdr1as was linked to the miR-7 target gene PIK3CD, suggesting that Cdr1as could function as a miR-7 inhibitor. The level of Cdr1as expression is highly correlated with HCC, a relationship that may be substantiated by considering all available information. Further validation is necessary to ascertain whether Cdr1as can function as a potential biomarker for HCC, as its role in this context remains unclear. Through circRNA microarray analysis, we identified the upregulation of circ 100338, which exhibited a robust association with shortened cumulative survival. Furthermore, a positive correlation was found between HBV infection and the progression of metastasis in patients with HCC (318). Circ_100338 acts as a sponge for miR-141-3p, which can be counteracted by miR-141-3p. This, in turn, hinders the progression of cell metastasis in liver cancer. By simultaneously expressing miR-141-3p and circ_100338 in MHCC97H cells, it is feasible to restore the suppression of invasive capacity caused by the excessive production of miR-141-3p. The overexpression of miR-141-3p can reverse the increased migratory and invasive ability of MHCC97H cells caused by circ_100338. These data indicate that circ_100338 could be used as a novel biomarker for diagnosing and assessing patient survival in patients with HBV-related HCC (318). A recent study has shown that the expression of circ_0067934, which is increased in HCC, can promote the Wnt/β-catenin signaling pathway by functioning as a miR-1324 sponge. MiR-1324 has the ability to impede the activation of the Wnt/β-catenin signaling pathway by specifically targeting the 3′-UTR of FZD5. Downregulating the expression of FZD5 and inhibiting the activation of the Wnt/β-catenin signaling pathway can substantially reduce the proliferation, migration, and invasion of Hep3B and HuH7 cells. This can be accomplished by upregulating the expression of miR-1324 or by downregulating the expression of circ_0067934. The study’s findings suggest that targeting the circ_0067934/miR-1324/FZD5/Wnt/β-catenin signaling axis could be a promising approach for treating HCC. Moreover, in this particular instance, cellular demise was seen (319, 320).

### 4.5 CircRNAs as diagnostic factors in HCC

The overall survival rate for people with HCC is dismal since the disease is typically detected at an advanced stage (321). Early-stage disease is characterized by a small number of symptoms and limited availability of biomarkers (322). Furthermore, the accuracy of the present biomarkers, like α-fetoprotein (AFP) and AFP-L3, in detecting lung cancer is only moderate (322). CircRNAs show high levels of abundance and stability in HCC tissue and bodily fluid, and they are tightly linked to many biological processes in HCC; hence, they have been suggested as possible diagnostic biomarkers for HCC. The ability of hsa_circ_0068669 to serve as a biomarker for the progression of HCC metastasis was demonstrated by Yao and colleagues. There was also a considerable correlation between the amount of microvascular invasion and the expression of hsa_circ_0068669 highlighted by them. The possibility of circRNAs as biomarkers for the diagnosis of HCC has been explored in a recent research (316, 323, 324). The upregulation of Circ-CDYL, a promoter for HCC, may lead to an increase in the expression of several proto-oncogenes. With an AUC of 0.64 [95% confidence interval (CI) = 0.55–0.72], Wei and colleagues (325) revealed the diagnostic performance of circ-CDYL in the early stage of HCC. This remark was made recently. After a thorough analysis of the levels of circ-CDYL expression in relation to HDGF and HIF1A, the results showed an improved diagnostic accuracy, as indicated by an AUC of 0.73 (95% CI = 0.65–0.80), a sensitivity of 75.36%, and a specificity of 66.67%. The AFP only showed an AUC of 0.59 (95% CI = 0.49–0.70), a sensitivity of 50.72%, and a specificity of 83.78% when compared to circ-CDYL with HDGF and HIF1A. According to the results of this experiment, it seems that the combination of circ-CDYL with HDGF and HIF1AN may serve as a more precise diagnostic biomarker compared to AFP. A research on the levels of expression of hsa_circ_0028502 and hsa_circ_0076251 was carried out by Jiang and colleagues (326) in malignant tissues and surrounding para-cancerous tissues. Based on consequences, it was observed that the rates of hsa_circ_0028502 and hsa_circ_0076251 were considerably lower in tissues that were correlated with HCC (*p* < 0.001). A noticeable correlation was discovered between the level of hsa_circ_0028502 and the stage of metastasis in the tumor nodes (*p* = 0.015), whereas the expression of hsa_circ_0076251 was associated with the stage of liver cancer at the Barcelona Clinic (*p* = 0.038). The AUCs of hsa_circ_0028502 and hsa_circ_0076251 were 0.675 and 0.738, respectively, when HCC tissues were differentiated from liver cirrhosis tissues and chronic hepatitis tissues. Additionally, Matboli and collaborators (327) evaluated the diagnostic performance of hsa_circ_001565, hsa_circ_000224, and hsa_circ_000520 for HCC. The results showed that when compared to AFP, these three hsa_circ_samples were more sensitive and specific. A diagnostic performance increase of 80% was achieved with the inclusion of these three biomarkers, leading to a sensitivity of 100% and a specificity of 83.33%. In an ROC curve study including 40 patients with HCC and 40 healthy controls, the AUC for hsa_circ_0016788 was found to be 0.851.59. This discovery expands upon what is already known. Additionally, the hsa_circ_0128298 gene, which was shown to be highly overexpressed in HCC tissues, had a sensitivity of 0.716, a specificity of 0.815, and an AUC of 0.668 (264).

**Figure 7:**
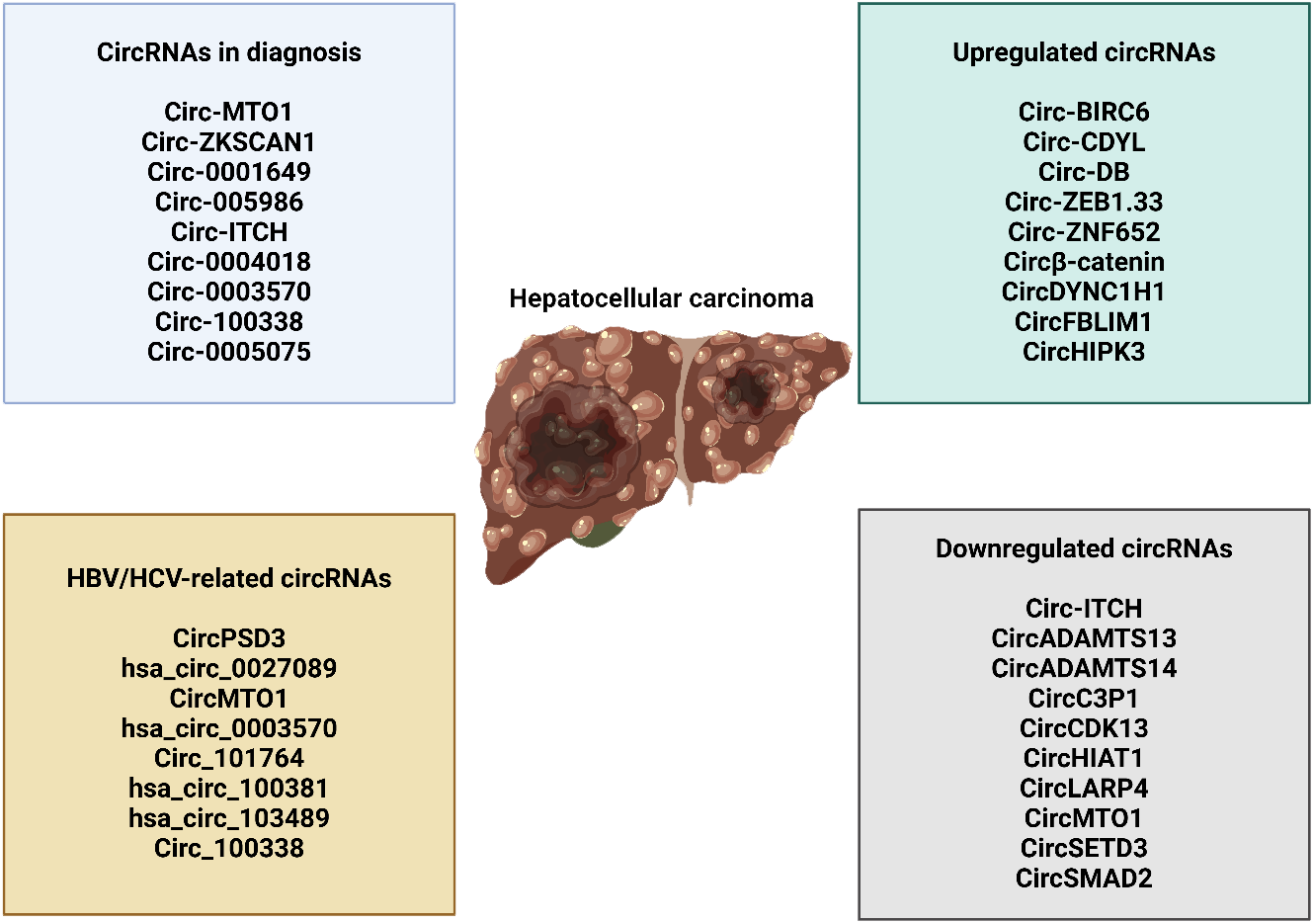
An overview of circRNAs in cancer diagnosis and therapy.

In addition, Yao and colleagues (328) conducted an analysis on the expression of circZKSCAN1 (hsa_circ_0001727) in a cohort of 102 patients who were diagnosed with HCC. The gene expression in tumor tissues was markedly lower compared to the non-tumorous samples that were matched to the tumor tissues (*p* < 0.05), as indicated by the results. The expression rate of circZKSCAN1 was represented to be remarkably associated with many clinical indicators, including tumor grade (*p* < 0.001), vascular invasion (*p* = 0.002), microvascular invasion (*p* = 0.002), the existence of cirrhosis (*p* = 0.031), and the number of tumors (*p* < 0.01). When used as a diagnostic biomarker, circZKSCAN1 consistently illustrated strong performance with an AUC of 0.834, a sensitivity of 82.2%, and a specificity of 72.4%. Plasma circRNAs can be utilized as diagnostic biomarkers for HCC, together with alterations observed in HCC tissue. In a comprehensive study conducted by Yu and co-workers (329), a plasma circRNA panel (circPanel) was developed and evaluated. This panel included three specific circRNAs: hsa_circ_0000976, hsa_circ_0007750, and hsa_circ_0139897. The main objective of this large-scale multicenter inquiry was to determine the effectiveness of this panel in identifying HCC that is connected to the HBV. The circPanel, a newly created diagnostic tool, outperformed AFP in terms of diagnostic performance in a validation sample of 306 individuals. The circPanel had an AUC of 0.843 (95% CI = 0.796–0.890), while AFP had an AUC of 0.747 (95% CI = 0.691–0.804). The circPanel demonstrated a reliable performance in diagnosing small HCC (solitary < 3 cm) and AFP-negative HCC compared to other approaches, with AUC values of 0.838 (95% CI = 0.776–0.900) and 0.857 (95% CI = 0.793–0.921), respectively. Thus, growing data emphasize that circRNAs play a significant role in the control of tumor formation and can be regarded as potential therapeutic and diagnostic factors in HCC (330–337). A clinical study has also shown that circ_0004001 can be utilized for the diagnosis of HCC (NCT06042842). **Figure 7** is an overview of circRNAs in HCC. **Table 1** is an overview of the ncRNAs in the regulation of tumorigenesis in HCC.

**Table 1:** The recent advances of ncRNAs in HCC.

Non-coding RNA	Remark	Reference
Circ_0004913	Inhibition of proliferation, metastasis, and glycolysis through absorbing miR-184	(338)
Circ_KIAA1429	Increasing tumorigenesis Upregulation of HMGA2	(339)
Circular RNA hsa_circ_0005218	Increasing early occurrence of hepatocellular carcinoma Binding to miR-31-5p to increase CDK1 levels	(340)
Hsa_circ_0010882	Increasing M2 polarization of macrophages	(341)
Circular RNA hsa_circ_0098181	Interaction with eEF2 to stimulate Hippo signaling for impairing invasion and migration of tumor cells	(342)
Circular RNA circ_0003028	Inhibition of miR-498 to increase ornithine decarboxylase 1 levels	(343)
Circ_0073228	Increase in the growth of tumor cells and suppression of apoptosis	(344)
Hsa_circ_001726	Sponging miR-671-5p to increase PRMT9 levels in enhancing invasion and metastasis	(345)
Hsa_circ_0000092	Binding to miR-338-3p to increase HN1 levels in tumorigenesis	(346)
Circ_MAPK9	miR-642b-3p inhibition to enhance STAT3 and LDHA levels	(347)
Circ_HMGCS1	Sponging miR-338-5p to increase IL-7 levels in cisplatin resistance	(348)
Circ-EIF3I	Inhibition of miR-361-3p to increase DUSP2 levels in enhancing tumorigenesis	(349)
CircZCCHC2 (hsa_circ_0000854)	Stimulation of the Rho/ROCk2 axis and the regulation of miR-936/BTBD7 to elevate tumorigenesis	(350)
LncRNA MIR4435-2HG	Increase in stemness through regulation of rRNA 2’-O methylation	(351)
LncRNA FAM99B	FAM99B can be derived from exosomes of mesenchymal stem cells to suppress tumorigenesis	(352)
LncRNA FAM13A-AS1	LncRNA FAM13A-AS1 is transcriptionally modulated by PHOX2B to increase PPARγ stability to control drug resistance	(353)
LncRNA SNHG16	The exosomal SNHG6 can enhance the metastasis of hepatocellular carcinoma through upregulating MMP9	(354)
LncRNA SLC2A1-DT	Positive interaction with c-Myc to increase carcinogenesis	(355)
LncRNA HEIH	Binding to miR-193a-5p to upregulate CDK8 to enhance growth and invasion	(356)
LncRNA RP11-620J15.3	Increase in glycolysis	(357)
LncRNA SNHG1	SNHG1 sponges miR-7-5p to increase IGF2BP2 levels	(358)
miR-612	miR-612 promotes RSL3-mediated ferroptosis	(359)
miR-375	miR-375 suppresses autophagy to disrupt sorafenib resistance	(360)
LINC00607	LINC00607 is induced by MYC and can promote tumorigenesis through miR-584-3p sponging and upregulating ROCK1	(361)
CircCPSF6	Sponging miR-145-5p to increase MAP4K4 levels	(362)
miR-26a	Tumor exosomes can deliver miR-26a to reduce lymphoid enhancer factor 1 levels in impairing tumorigenesis	(363)
CircTMEM181	Upregulation of ARHGAP29 through inhibition of miR-519a-5p to impair metastasis	(364)
Hsa_circ_0129047	Sponging miR-492 to increase LYVE1 levels in impairing tumorigenesis	(365)
Circ-SNX27	Accelerating tumorigenesis through sponging the miR-375/RPN1 axis	(366)
Hsa_circ_0000098	Sponging miR-136-5p to increase MMP2 levels	(367)

## 5 Conclusion, future perspectives, and challenges

The diverse biomolecular properties of HCC are evident in its wide range of clinical outcomes and progression, as well as its morphological features, which are evaluated by radiologists and pathologists (368). The foundation of both cirrhotic nodules and hepatocarcinogenesis is controlled by different molecular mechanisms. This development is linked to the up- or downregulation of many miRNAs. The current research illustrates that mTOR is the most frequently observed miRNA-regulated pathway in HCC. Moreover, the rates of miRNA expression also modulate other pathways, such as apoptosis, Wnt, and MAPK. In addition, there are other miRNAs that are expected to have therapeutic significance, particularly in relation to post-surgical recurrence and responsiveness to systemic therapy. These miRNAs are involved in at least one of the main molecular pathways that contribute to the development of hepatocarcinogenesis. The identification of specific tissue and serum miRNAs that can accurately predict the development of HCC in patients with cirrhosis, anticipate the recurrence of HCC after surgery, or forecast the response to systemic treatment has the potential to greatly enhance the management of these patients. The clinical application of miRNAs has consistently posed challenges, mostly due to the variation among laboratories and the identification of an appropriate control for normalization. A recent study conducted by our team revealed the alteration in the miRNA profile of HCC compared to a group of healthy livers and a group of cirrhotic tissues, which were used as non-tumor controls. Some authors have proposed utilizing stable miRNAs as controls when investigating the expression of other miRNAs. In order to gain a deeper understanding of the potential role of these molecules as theragnostic indicators in HCC, it would be beneficial to provide further details regarding the most commonly observed miRNA in live animal models, such as rats and mice. Based on the existing knowledge, this would be carried out in a manner that is appropriate and in line with the current circumstances. A diagnostic method for liver nodules based on miRNA and a treatment that regulates miRNA are still being developed. However, it is necessary to address the issue by establishing standardized protocols for miRNA analysis in different laboratories. These two approaches of diagnosis and treatment are essential.

The primary goals of this review are to provide a foundational understanding of liver cancer genesis, to discuss the role of lncRNA in this process, to examine the impact of tumor angiogenesis on HCC progression, and to explain how lncRNA controls tumor angiogenesis (369). Investigating how lncRNAs regulate tumor cell phenotypic alterations that promote HCC cell invasion and metastasis was the next step. Once in the circulation, lncRNA helps HCC cells avoid detection by the immune system by changing how susceptible tumor cells are to anoikis and by giving the immune system a way to avoid cancer cells in general. Finally, we will go over two questions that still have not been answered regarding cancer metastasis: targeted metastasis and tumor dormancy. These two matters hold a lot of potential. Additionally, the pro-metastasis niche, vascular dormancy, and immunological dormancy are the conventional explanations for these two inquiries. A lot of questions remain unanswered, despite the fact that new research has shed light on the role that lncRNA plays in HCC metastasis. Even though the best way to treat HCC is to remove the tumor surgically when it is still small, most individuals are found to have the disease far advanced when they have surgery. The good news is that there are now very few medications that can be used as a first line of defense against HCC. The multi-targeted tyrosine kinase inhibitors doxorubicin and sorafenib are two such examples of such drugs. It was found that many essential signaling pathways are involved at the start of HCC. The MEK pathway, the PI3K/AKT/mTOR system, the Wnt/β-catenin pathway, the JAK/STAT route, and the IGF pathway are all pathways that fall under this category (370). There is evidence suggesting that lncRNAs play a role in controlling the signal pathway mentioned earlier and offer new strategies for the clinical diagnosis and treatment of HCC (223). There has been substantial progress in tumor immunotherapy in recent years, leading to the widespread use of tumor immunovaccine in clinical settings. HCC is a common tumor that is linked to inflammation, and immunotherapy for this specific malignancy has reached an advanced stage. Tremelimumab, a CTLA-4 inhibitor, pembrolizumab, a PD-1/PD-L1 inhibitor, and nivolumab have all been used in clinical therapy (371). A recent study has shown that lncRNAs play a role in regulating the immune system in cancer. Special attention should be given to the investigation of immunotherapy that is linked to lncRNA in future investigations. Furthermore, the investigation of focused spread of cancerous growths is a captivating and auspicious research pursuit. The preferred sites for the spread of different types of tumors are unique from each other. Enhancing our comprehension of the underlying mechanisms that trigger the dissemination of tumor metastasis to particular sites may facilitate the amelioration of their treatment. The phenomenon of tumor dormancy has attracted growing attention, leading to several experimental investigations. Conversely, the study of tumor dormancy is currently in its initial phases, and numerous crucial inquiries remain unresolved. Is it accurate to say that all cancers in the human body contain dormant tumor cells? What is the duration of tumor dormancy? Which types of cancer cells are in a state of dormancy? Can latent tumor cells always become active? What is the method for reviving them? Ultimately, future research on lncRNAs should prioritize investigating their role in modulating the immune system, targeting metastasis, and regulating dormancy in liver cancer, to comprehend its role in metastasis and as a promising target for pharmaceutical intervention.

Emerging evidence indicates that some circRNAs show varying levels of expression in the tissues of patients with HCC. Furthermore, the disruption of these circRNAs is linked to clinicopathological features in patients with HCC (372). CircRNAs play a regulatory role in transcription by acting as sponges for miRNAs and RBPs. This enables them to control the production of certain target proteins and miRNAs. miRNAs and proteins contribute to the advancement of HCC and are linked to cell invasion, metastasis, and proliferation. The unique benefits of circulatory RNAs, including their high quantity, durability, and occurrence in many physiological fluids, render them appealing as diagnostic and prognostic markers and as targets for therapy in cases of HCC. circRNAs are very stable and plentiful in exosomes generated from serum. Exosomes carry a cargo of RNAs and proteins that tumor cells release and transfer to other cells in order to affect their behavior. Hence, circRNAs can play a dual role in promoting cancer advancement and serving as non-invasive biomarkers for cancer detection. Moreover, the presence of fusion circRNA contributes to the cancer cells’ capacity to withstand treatment. This resistance is a major obstacle in the use of chemotherapy for cancer treatment, and it could be a contributing factor to the ineffectiveness of current treatments in eradicating malignant tumors. However, it remains uncertain whether any circRNA, including fusion circRNA, is involved in the resistance of HCC to treatment. The investigation of circRNAs in HCC is still in its early stages, unlike the research on coding RNAs, mRNAs, and lncRNAs. Currently, there is a lack of knowledge about the prevalence of functional circRNAs in HCC. The majority of these studies have primarily examined the functions of these circRNAs as miRNA sponges in promoting the proliferation of HCC cells. However, only a limited number of these circRNAs have been thoroughly researched in terms of their mechanisms of action. A comprehensive understanding of the synthesis, degradation, cellular localization, functions, and mechanisms of action of the vast majority of circRNAs that are currently awaiting investigation is still lacking. A more comprehensive study of the relationship between circRNAs and the causes, behaviors, and molecular processes of HCC would enhance our understanding of human disorders. On top of that, this would provide new opportunities for improving the diagnosis, prognosis, and treatment of HCC. During hypoxia, the exosomes can enhance the progression of lung pre-metastatic niche in HCC through the delivery of miR-4508. The upregulation of exosomal miR-4508 is observed in HCC, and it can modulate the p38 MAPK–NF-κB axis (373).

The current studies highlighted the fact that dysregulation of ncRNAs commonly occurs in HCC. The major ncRNAs including miRNAs, lncRNAs, and circRNAs are therapeutic, diagnostic, and prognostic factors. From a therapeutic aspect, the ncRNAs can be targeted by the drugs in HCC therapy. However, the drugs have a poor pharmacokinetic profile and their clinical importance is limited. Therefore, the researchers are suggested to focus on the application of genetic tools for the regulation of ncRNAs. Moreover, the epigenetic drugs have a low ability in binding to the ncRNAs as epigenetic factors and regulate cancer progression. Although a high number of studies have evaluated the function of ncRNAs in the modulation of tumorigenesis, one of the largest limitations is the lack of focus on the application of nanoparticles for the regulation of ncRNAs.

## Author contributions

YT: Validation, Software, Project administration, Methodology, Investigation, Formal Analysis, Data curation, Conceptualization, Writing – review & editing, Writing – original draft. MZ: Writing – review & editing, Writing – original draft, Visualization, Supervision, Resources, Methodology, Formal Analysis, Data curation, Conceptualization. L-XL: Writing – original draft, Supervision, Project administration, Methodology, Investigation, Formal Analysis, Data curation, Conceptualization. Z-CW: Writing – review & editing, Validation, Software, Methodology, Investigation, Data curation. BL: Writing – review & editing, Visualization, Validation, Resources, Project administration, Methodology, Investigation, Formal Analysis, Data curation. YH: Writing – original draft, Project administration, Methodology, Data curation, Conceptualization. XW: Writing – review & editing, Validation, Supervision, Project administration, Methodology, Investigation, Formal Analysis, Data curation. Y-ZL: Writing – review & editing, Validation, Project administration, Methodology, Investigation, Formal Analysis, Data curation. FW: Writing – review & editing, Writing – original draft, Software, Methodology, Investigation, Formal Analysis, Data curation, Conceptualization. XF: Writing – review & editing, Writing – original draft, Supervision, Project administration, Methodology, Data curation. YYT: Writing – review & editing, Writing – original draft, Visualization, Supervision, Resources, Project administration, Methodology, Investigation, Funding acquisition, Formal Analysis, Data curation.
